# Humidity Sensors Principle, Mechanism, and Fabrication Technologies: A Comprehensive Review

**DOI:** 10.3390/s140507881

**Published:** 2014-04-30

**Authors:** Hamid Farahani, Rahman Wagiran, Mohd Nizar Hamidon

**Affiliations:** 1 Department of Electrical and Electronic Engineering, Faculty of Engineering, Universiti Putra Malaysia, Serdang, Selangor 43400, Malaysia; E-Mail: rwagiran@upm.edu.my; 2 Functional Devices Laboratory, Institute of Advanced Technology, Universiti Putra Malaysia, Serdang, Selangor 43400, Malaysia; E-Mail: mnh@upm.edu.my

**Keywords:** humidity sensors, capacitive/resistive sensor, relative humidity, fabrication technologies, humidity sensing properties, nanocomposites, ceramic/polymer, perovskite materials, protonic conduction mechanism, thick/thin film

## Abstract

Humidity measurement is one of the most significant issues in various areas of applications such as instrumentation, automated systems, agriculture, climatology and GIS. Numerous sorts of humidity sensors fabricated and developed for industrial and laboratory applications are reviewed and presented in this article. The survey frequently concentrates on the RH sensors based upon their organic and inorganic functional materials, e.g., porous ceramics (semiconductors), polymers, ceramic/polymer and electrolytes, as well as conduction mechanism and fabrication technologies. A significant aim of this review is to provide a distinct categorization pursuant to state of the art humidity sensor types, principles of work, sensing substances, transduction mechanisms, and production technologies. Furthermore, performance characteristics of the different humidity sensors such as electrical and statistical data will be detailed and gives an added value to the report. By comparison of overall prospects of the sensors it was revealed that there are still drawbacks as to efficiency of sensing elements and conduction values. The flexibility offered by thick film and thin film processes either in the preparation of materials or in the choice of shape and size of the sensor structure provides advantages over other technologies. These ceramic sensors show faster response than other types.

## 1. Introduction

In recent years improvements in sensor manufacturing technologies have occurred driven by post-process high-speed, low-power and low-cost microelectronic hybrid circuits [1–4], modern signal conditioning methods [5,6] and advances in miniaturization technologies [7–11]. The requirement for commercial competiveness is sequential enhancement of quality and product reliability. Furthermore, it is important to know the degree of efficiency of each sensor related to its calibration circumstances and sensing mechanism [12]. Today, simulation techniques and design aides are adequately used to predict and improve output data prior to implementation of mass production processes to save time and enhance quality [13,14]. Miniaturization of sensor devices offer numerous advantages such as low hysteresis [15], batch fabrication [16], ease of packaging/integration along with the corresponding cost reductions [17–20].

It is known that humidity plays a significant role in every part of the Earth in biology and automated industrial processes. To have a desirable surrounding atmosphere, it is essential to monitor, detect and control the ambient humidity under different conditions ranging from low temperature to high or in mixtures with other gases by precise and provident sensors [21,22]. Utilization in intelligent systems and networks as monitoring sensors to determine the soil moisture during irrigation in agriculture, or for diagnosis of corrosion and erosion in infrastructures and civil engineering are among the applications of humidity sensors [23]. In fact, the need for protection of environmental conditions has been leading to extensions in various humidity sensor developments based on the use of physical and chemical methods in presence of organic, inorganic or hybrid materials [24–27]. Advancement of humidity sensory systems encompasses enhanced efforts in betterment of transducer performance such as sensing elements [28–30], structure design [31,32], principle of mechanism [33,34], and fabrication technologies [35–37]. In this context the transducer materials are the key features, followed by the availability of suitable manufacturing technologies, free choice of device geometrical properties to attain the required dimensional efficiencies, optimisation of surface for the occurrence of conductance, ease of production flow and investment expenses.

This paper reviews reported studies on different humidity sensor fabrication technologies, principles of materials, applications, and moisture sensing mechanisms. Further, recent developments of dampness-sensitive materials, *i.e.*, composite or single-species in nano-scale processing system are among the principal considerations. Synthesis and preparation of metal oxides as ceramic type hygrometers and film based humidity sensors are further assessed. To provide a clear comprehension of operation principles, the universally observed sensing mechanism of porous matters which is of an ionic type, will be detailed. Nanocomposites incorporating ceramics, ceramics/polymers and polymers/carbon nanotubes with nanoporous, nanofiber and nanowire forms are amongst the most promising materials for future applications, and their preparation processes deserve mention.

The rest of this article is organized as follows: in Section 2, the fundamental definitions of humidity and the procedures for parameter measurement are described. Then, a classification of humidity sensors and the related applications are given in Section 3. The principle of ceramic humidity sensor operation based upon hydrogen permeation and water protonation is detailed in Section 4. Whilst Section 5 provides a survey of impedance type sensors, Section 6 covers capacitive type transducers. A summary of the review is given in Section 7.

## 2. Humidity Basics and Measurement Parameters

Humidity is defined as the amount of water vapour in an atmosphere of air or other gases. Humidity parameters are stated in diverse ways and the corresponding units are based on the measurement technique used. The most commonly used terms are “Relative Humidity (RH)”, “Parts Per Million (PPM)” by weight or by volume and “Dew/Frost Point (D/F PT)”, in which the two latter are subclasses of “Absolute Humidity (AB)”. Absolute Humidity units are applicable for the primary measurement results inasmuch as one is able to directly measure the value of the water vapour content. In contrast, Relative Humidity is true for the secondary measurement results, since measurement of the water vapour values is mediated in some fashion.

Absolute Humidity (vapour density) is defined as a ratio of the mass of water vapour in air to the volume of air, with the unit of grams per cubic meter or grains per cubic foot (1 grain = ^1^/_7000_ pound lb) and expressed as:

(1) $$AB=\frac{m_{w}}{v}$$

where *AB* is the absolute humidity (g/m^3^ or grains/ft^3^), *m_w_* is the mass of water vapour (gram or grain) and *v* is the volume of air (m^3^ or ft^3^).

Relative Humidity (abbreviated as RH) is defined as ratio of the amount of moisture content of air to the maximum (saturated) moisture level that the air can hold at a same given temperature and pressure of the gas. RH is a temperature dependent magnitude, and hence it is a relative measurement. The RH measurement is stated as a percentage and determined by the expression:

(2) $$RH\%=\frac{P_{V}}{P_{S}}\times 100$$

where *P_V_* is the actual partial pressure of moisture content in air and *P_S_* is the saturated pressure of moist air at the same given temperature (both in Bar or KPa).

Saturation Humidity is defined as the ratio of the mass of water vapour at saturation to the volume of air:

(3) $$SH=\frac{m_{ws}}{v}$$

where *SH* is the saturation humidity (g/m^3^), *m_ws_* is mass of water vapour at saturation (g) and *v* is the volume of air (m^3^). The saturation humidity is a function of temperature and can provide the maximum amount of moisture content (mass) in a unit volume of gas at a given temperature.

According to Equation (3), Relative Humidity can be represented in other way by calculating the ratio of absolute humidity to saturation humidity as a percentage as follows:

(4) $$RH\%=\frac{AB}{SH}\times 100$$

Parts Per Million by volume (PPMv) is defined as volume of water vapour content per volume of dry gas, and Parts Per Million by weight (PPMw) is obtained by multiplying PPMv by the mole weight of water per mole weight of that gas or air. PPMv and PPMw are among the absolute humidity measurements.

Dew point is defined as a temperature (above 0 °C) at which the water vapour content of the gas begins to condense into liquid water, and Frost point is the temperature (below 0 °C) at which the water vapour in a gas condenses into ice. D/F point parameters are functions of the pressure of the gas, but independent of temperature and are amongst the absolute humidity measurements. In other words, dew point is the temperature at which the saturation water vapour pressure is equal to the partial pressure of the water vapour (in an air atmosphere). The difference between the ambient temperature and the dew point temperature is a measure of the ambient relative humidity [38,39].

## 3. Humidity Sensors Classification and Applications

Humidity sensing studies have progressed rapidly and humidity sensors—regardless of fabrication technique—have been widely employed in industrial and household applications as instrumentation equipment or for human comfort issues. Due to the different operating conditions of moisture sensors in different areas of application ranging from indoor to open air uses, various types of humidity sensing instruments have been developed based on different work principles and diverse hygroscopic sensing materials [40–45]. Amongst the various humidity evaluation terms and units, absolute humidity and relative humidity are the most prevalent. Based on the units of measurement, humidity sensors are subsumed in two main classes: “Relative Humidity (RH)” and “Absolute Humidity” sensors (hygrometers). In the majority of humidity measurement applications relative humidity measurements are more preferable than absolute humidity ones. RH% is most commonly used because is generally simpler and thus cheaper and is extensively applied in applications involving indoor air quality and human comfort issues [46]. Accordingly, in research laboratories and public applications, RH is ubiquitously applied to simplify the design process and further use as a secondary sensor. Absolute humidity units, namely Dew/Frost point (D/F PT) and Parts Per Million by weight (PPMw) or by volume (PPMv) are mostly used for traceable purposes (trace moisture measurement) as primary sensors. As absolute units they describe the absolute amount of water vapour in gaseous environments.

As most of the commercially available humidity sensors in use are relative humidity sensors, these can be grouped based upon their sensing material types and according to their operating principle. In the 1980s the different sensing elements were proximately classified into three main groups of electrolytes, porous ceramics and organic polymers conforming to the Yamazoe and Shimizu classification [47]. Around ten years later, in 1994, according to Traversa's classification, commercially developed humidity sensors were predominantly based on the porous ceramics and organic polymer films [48]. In 2005, another type of the categorization was provided by Chen and Lu, in which the Relative Humidity sensors were divided into three classes: ceramic, semiconductor and organic polymer types. Moreover, in this classification Absolute Humidity sensors were available in two types: solid moisture and mirror-based (chilled mirror) sensors [49].

At the present time, plenty of different brand humidity sensors on the market or under development in laboratory studies are RH sensors, which are further categorized into three classes, including ceramic type (semiconductor), organic polymer-based sensors, and organic/inorganic hybrid sensors (polymer/ceramic). By reviewing the published results in this field, the authors have determined that well-nigh 80% of these three types are based on the electrolytic properties of the sensing matter, by virtue of the inner water electrolytes. Humidity sensors using nanowires, nanofibers, nanorods and p-n heterojunctions are subclasses of the ceramic (inorganic) type. Regarding the intrinsic properties of sensing elements, ceramic types can be designed by utilizing either semiconducting or dielectric metal oxide composites. Furthermore, polymeric types in turn can be based on conducting or non-conducting (dielectric) polyelectrolytes.

All three categories of such sensors (so-called hygrometric sensors), utilize changes in the physical and electrical properties of the sensitive elements when exposed to the different atmospheric humidity conditions of the surrounding environment, and provide a measure of the humidity due to some amount of adsorption and desorption of water vapour molecules. It has been found that porous films exhibit higher humidity sensitivity than the nonporous counterparts [50]. The presence of intergranular or intragranular porosity as well as pore size distribution are also among the determinative factors for humidity transducers [51]. Humidity measurement in the hygrometer type of sensors is accomplished by measurement of either the electrical impedance (conductance) or capacitance of the sensing matters which is proportional with the change of some organic or inorganic synthetic body's physics. The basis of the moisture sensing is the physical and chemical adsorption of water molecules that will be further described in the next section.

In 1937 a electrolytic humidity sensor based on lithium chloride (LiCl) developed by Dunmore [52], became the first and only electrical moisture sensor available until around the middle of the 1970s. LiCl electrolyte sensors have been extensively utilised in radiosonde (weather balloons that are used to measure atmospheric parameters) circuit applications as well as medical uses. A porous supporting material was immersed in a humidity sensitive partially hydrolysed polyvinyl acetate which was impregnated with LiCl solution and a potential difference was applied across the supports to form an electrolytic cell. By absorbing the atmospheric water vapours via the porous medium, the ionic conductivity of the cells were changed and the amount of humidity detected. Since these devices exhibited low response/recovery times and suffered from the incapability to work under very moist conditions or in the proximity of various solvents, the development of humidity sensors based upon other materials was started to replace electrolyte-based humidity sensors.

Impedance-sensitive type humidity sensors which encompass all three classes, are further classified into ionic and electronic (charge carriers) conduction type sensor devices. This classification is determined by the mechanism of the electrical transport. Electrolyte-based sensors being special cases that only follow the ionic transport mechanism. Later we present and classify the different types of sensing materials, working principles (impedance ionic or impedance electronic or capacitance type) with humidity and temperature ranges of various hygrometer sensors.

Traditionally, the organic polymer film humidity sensors fall into the fundamental categories of resistive type (impedance type) [53,54] and capacitive types [55], the former being further subdivided to electronic and ionic conduction type sensors. This classification is based upon the sensing mechanism in which the prior one contains polyelectrolytes respond to water vapour variations by changing their resistivity, while in the latter humidity is measured based on the variation of the dielectric constant of the polymer dielectrics and hence changes in capacitance.

Ceramic type humidity sensors based on metal oxides have exhibited some superior advantages in comparison to polymer films from the viewpoints of their mechanical strength, thermal capability, physical stability and their resistance to chemical attack, which reveals them to be the most promising materials for electrochemical humidity sensor applications [48]. This class of sensors could be divided into two groups, impedance or capacitive types, according to their sensing mechanisms, based on whether they utilise either the conductance or capacitance properties of the sensing layer for detection of humidity [56,57]. The impedance type sensors in turn are subdivided into ionic-conduction [58,59] and electronic-conduction [60,61] types. The electronic and ionic types provide the value of moisture by quantifying the changes of conductivity of the sensing films versus different levels of humidity. The p-n heterojunction humidity sensors are also categorized among the ceramic types.

Basically, metal-oxide ceramics used in humidity sensor applications are prepared by conventional [62] and advanced [63] ceramic processing methods and are mainly developed to offer porous bodies [64–66]. The advantage of an absorbent spongiform surface rather than a condensate is a greater permeability of the water molecules, so water vapour molecules can easily pass through the pore openings and capillary condensation occurs in the capillary porous structures which were formed between the grain distributions in ceramic surface during the pore removal process [48]. The state of the art in development and prospect challenges of humidity sensors are provided in Table 1. Furthermore, the Table gives the humidity sensor classification based on transduction technologies and sensing materials. Table 2 explains and summarizes the various types of reviewed humidity sensors based on their fabrication technologies, sensing materials, working principles and operating mechanisms.

## 4. Working Principle of Protonic-Conduction Type Ceramic Humidity Sensors

Porous ceramic sensors are extensively used in industry and research laboratories. The unique structures of ceramic materials comprising grains, grain boundaries, surface areas and controlled porous microstructures, makes them suitable candidates for electrochemical sensor applications [67,68]. Various humidity sensing mechanisms have been proposed and studied for different dampness-sensitive reported ceramics. The mechanism principle of all the ceramic humidity sensors, *i.e.*, ionic conduction, electronic conduction, solid-electrolyte and capacitive type, relies on the superficial water vapour adsorption based on chemical adsorption (chemisorption), physical adsorption (physisorption) and capillary condensation processes [69]. Most of the currently available humidity sensors are constructed based on a porous sintered body structure ceramics and utilize the ionic type humidity-sensing principle. By water adsorption on the ceramic surfaces, their electrical properties would change and this change encompasses the resistance, capacitance or electrolytic conduction depending upon the sensor type [48]. In ionic type sensing elements, by increasing the humidity, the conductivity increases and thus the dielectric constant increases [70,71].

### 4.1. Hydrogen (H^+^) Ions Diffusion

**Figure 1.**
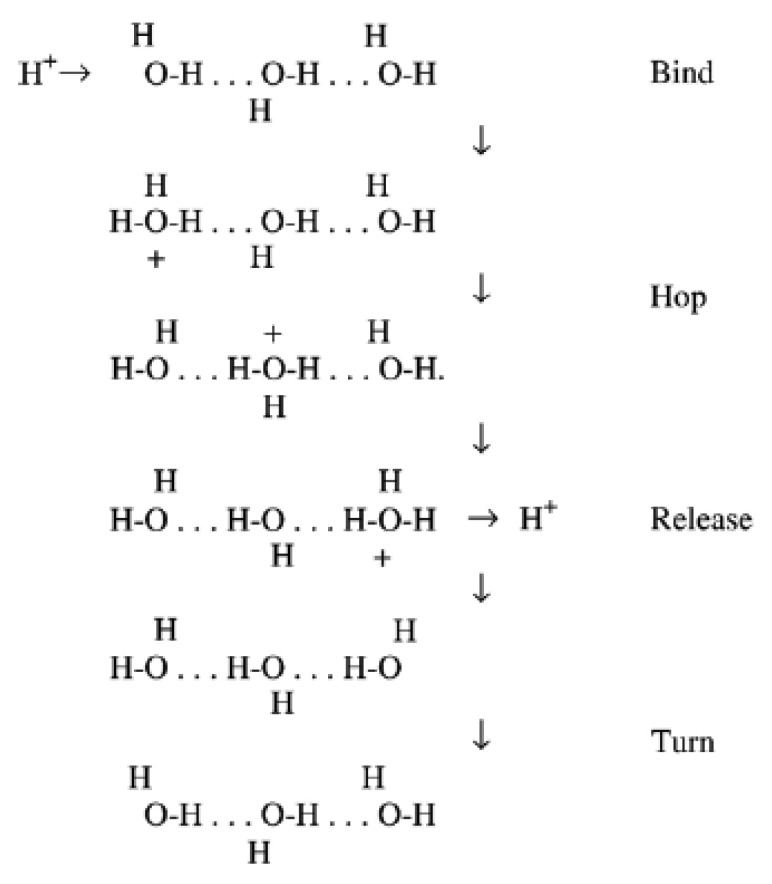
Proton conduction of the H-bonded networks between water molecules [74]. The express substitution of hydrogen (H^+^) between hydronium cation (H_3_O^+^) and water molecule (H_2_O) without diffusive action of an individual hydrogen or oxygen atom.

The transmembrane intrinsic energy of the hydrogen ion plays a substantial role in electrochemistry, e.g., in humidity sensing transduction. In an equilibrium condition the proton mobility of water and its derivatives (*i.e.*, vapour) is pretty high (abnormal) [72], and further establishment of aqueous clusters promote the proton concentration of the surface/bulk across water molecules. The prevalent justification of experimental data concentrated on the dynamics of the proton transfer process from site to site (prototropic mobility). This unique mechanism was proposed around 200 years ago in 1806 by Theodor von Grotthuss [73]. The Grotthuss mechanism was acknowledged during the aquatic electrolysis of water for the positive charge migrations. In this conduction mechanism, which exists in all liquid water, protons are tunnelled (proton dancing) from one water vapour molecule to the subsequent one through hydrogen bonding, as shown in Figure 1.

In the water uptake proton-carrier mechanism of ceramic humidity sensors, the adsorbed water is condensed on the material surface/bulk and conduction will be carried out by the protons (electron-accepting part of the water molecules) in superficial liquid-like layers (aqueous layers). In fact the protons are the dominant carrier responsible for electrical conductivity when exposed to humidity.

### 4.2. Diffusion and Mobility of Hydroxide (OH^−^) Ions

It is universally suggested that protons (H^+^) and hydroxide ions (OH^−^) are quickly diffused due to surface collision or self-ionization of water molecules, and this leads to initial separation of (H^+^, OH^−^) ions as:

$${\text{H}}_{2}\text{O}<=>{\text{H}}^{+}+{\text{OH}}^{-}$$

**Figure 2.**
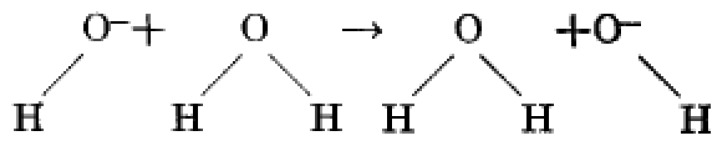
Proton transfer mechanism of hydroxide ions [75].

A similar transportation mechanism to that of hydrogen ions was put forward for hydroxide ions, called the Grotthuss hydroxide transfer. In agreement with Conway *et al.* the mobility of hydroxide ions can also occur via a proton transfer mechanism [75], as shown in Figure 2.

Due to the amphoteric nature of water, and hence auto ionization reaction of water vapour on a surface, a H_2_O molecule loses nucleus of one of its hydrogen, H^+^, atoms to become a hydroxide ion, OH^−^. The released hydrogen nucleus immediately protonates another H_2_O molecule to form an oxonium (hydronium) ion, H_3_O^+^. This is a simplest theory of water vapour interaction with a surface and is exemplified as follows:

$${\text{H}}_{2}\text{O}+{\text{H}}_{2}\text{O}<=>{\text{H}}_{3}{\text{O}}^{+}+{\text{OH}}^{-}$$

In environments with low humidity levels, when the sensor surface is slightly covered by water vapour molecules and only dissociated hydroxyl functional groups are present, the charge carriers are protons (hydrogen ions) and proton migration proceeds through hydrogen hopping between adjacent hydroxide ion sites. At higher humidity values, when a continuous water layer is formed charge transfer still arises by proton hopping between neighbouring sites of water vapour molecules. Formation of more water layers facilitates the dissociation to produce hydronium groups. Easy dissociation of physisorbed multi water layers could be along the high electrostatic fields in the chemisorbed layer. When surface coverage with water molecules is complete, diffusion of H_3_O^+^ on hydroxide ions is dominant, but H^+^ transfer by OH^−^ between neighbouring water molecules also occurs. The charge carrying continues when H_3_O^+^ transfers (releases) a proton to a neighbouring H_2_O molecule and forms another H_3_O^+^. The procedure is carried out by dancing of protons (hydrogen ions) from one water vapour molecule to another, thereby leading to a remarkable change of resistance and capacitance.

### 4.3. Water-Adsorption and Conduction Mechanism on Ceramic Oxide Solid Surfaces

The principle of adsorption, *ergo* the dissociation mechanism of H_2_O on activated metal oxide sites has received abundant attention as early as the 1960s [76]. In the study on surface behaviour and existence of hydroxyl functional groups, the infrared spectroscopy technique was frequently applied by several researchers to disks of iron oxide and silica [70,77]. Similarly, the conduction principle of water vapour sensing on the transducer surface (electron donation, protonic transport, or interposition of both) was not yet known until around the mid-1960s. Nonetheless, the reality was obvious that the conductivity—along of self-ionization, water electrolytic properties, diffusion, and reorientation of protonic species—is overcome by load transport on the surface and probably is simpler than in the bulk [78]. Moreover, the hydration susceptibility of inorganic surfaces could be greater than the organic or internal ones, thereby facilitating dissociation and protonation [79].

**Figure 3.**
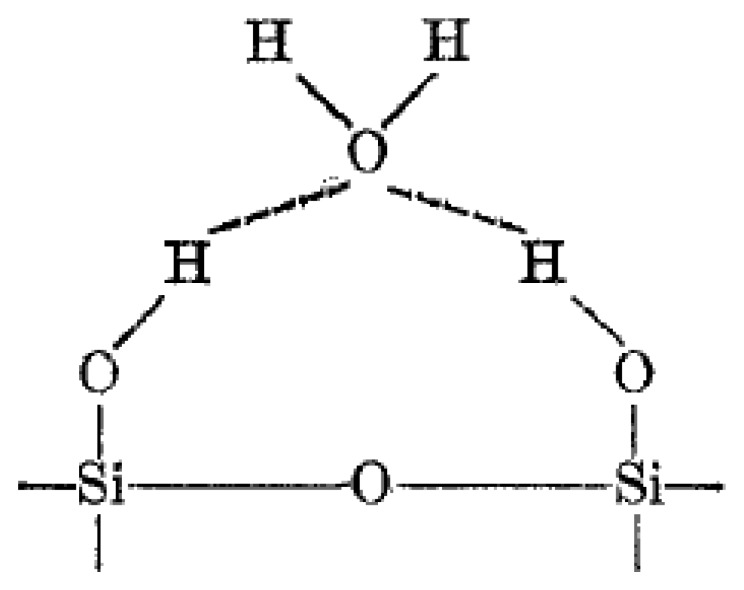
Adsorption sites on the silica surfaces, and formation of hydroxyl pairs to hold water molecules [87].

The mechanism of water molecule interaction on metal oxide surfaces, hence aqua protonation and ionic-conduction in ionic space charge layers was discovered and explained in studies of silica gel and hematite (α-Fe_2_O_3_) from about 1968 to 1971 [80–83]. The surface of most metal oxides is covered with hydroxyl groups when exposed to humid atmospheres (Figure 3), thus hydrogen bonding forms to further adsorb water molecules [84,85]. Normally, porous ceramics possess a very small surface area compared with powders that are nonporous or have low degrees of porosity. However when very low amounts of water are adsorbed in porous ceramics, their electrical conductivity is remarkably changed with the variation of water vapour adsorption [86].

**Figure 4.**
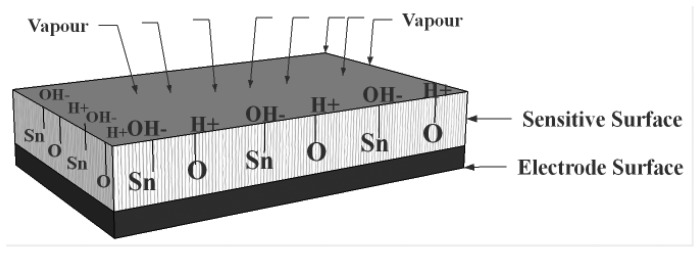
Illustration of water vapour chemisorption and hydroxyl layers on the surface of SnO_2_.

When ceramic oxides are exposed to atmospheric moist air, in the first stage of the interaction a few water vapour molecules are chemically adsorbed (chemisorption) at the neck of the crystalline grains on activated sites of the surface, which is accompanied with a dissociative mechanism of vapour molecules to form hydroxyl groups (two hydroxyl ions per water molecule). As an interaction between the surface ions of the grain necks and the adsorbed water, the hydroxyl group of each water molecule is adsorbed on metal cations which are present in the grains' surfaces and possess high charge carrier density and strong electrostatic fields, thus providing mobile protons. The protons migrate from site to site on the surface and react with the neighbour surface O^2−^ groups (oxygen) to form a second hydroxyl (OH^−^) group [86]. Figure 4 shows the interfacial adsorption and hydroxyl coverage of SnO_2_ [88]. The chemisorbed layer is the first formed layer so once it has formed on the surface it will not change further by exposure to humid air.

As a second stage, after chemical completion of the first layer, subsequent water vapour layers are physically adsorbed (physisorption) on the first formed hydroxyl layer, and stack to form the physisorbed hydroxyl multilayer. After forming the first physisorbed layer, another water molecule adsorbs via double hydrogen bonding to two neighbouring hydroxyl groups. As water vapour continues to increase in the surface, an extra layer forms on the first physisorbed layer, therefore the physisorption changes from monolayer to multilayer. These layers are less ordered than the first physisorbed layer, and water vapour molecules in these layers may be only singly bonded to local hydrogens.

**Figure 5.**
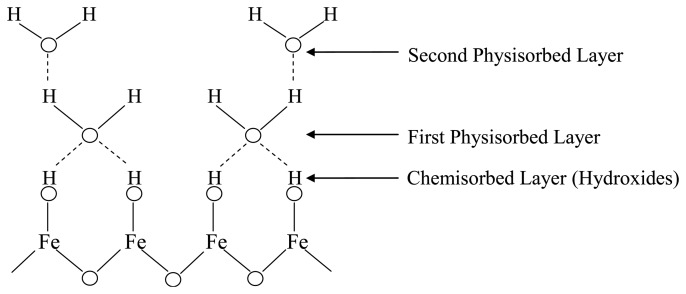
Multilayer structure of adsorbed water vapour molecules on the surface of iron oxide [83].

Finally, by forming the more layers, a large amount of water molecules is physisorbed on the necks and flat surfaces, hence singly bonded water vapour molecules become mobile and able to form continuous dipoles and electrolyte layers between the electrodes, resulting in an increased dielectric constant and bulk conductivity [89]. Therefore, the slight variations of conductivity with humidity adsorption can be due to a water protonation and protonic conduction mechanism on the surface. Multilayer formation due to water vapour physisorption can be certified by observing the increase of dielectric permittivity [86]. Figure 5 shows multilayer structure of adsorbed water vapour molecules on the surface of iron oxide [83]. A similar mechanism of hydroxylation, and hence multilayer formation on the surface of silica, has been reported by Hair and Hertl in 1969 [82].

Physisorption of water vapour molecules can only be accomplished at temperatures below 100 °C, while at higher temperatures up to about 400 °C, only chemisorption is responsible for the surface interaction of hydroxyls in ceramics [48].

The porous structure of ceramics plays a decisive role in the interactions and physisorption of water vapour molecules in ceramic type humidity sensors [90,91]. The humidity easily adsorbs throughout the open porosities and leads to water condensation within the capillary pores which are distributed between the grains. The water condensation tends to take place on the neck of the grain surfaces, and the amount of condensed water is mostly dependent on the open pores volume, pore radius sizes and distributions. If there are cylindrical pores in the specimen, the pore radius at which capillary condensation beings to take place can be evaluated based on the Kelvin equation [92] as follows:

(5) $$r_{k}=\frac{2\gamma M}{\rho RT\ln(p_{s}/p)}$$

where *r_k_* is the Kelvin radius of the cylindrical open pores (open at both ends), γ, *M* and ρ are the surface tension (72.75 dyn/cm at 20 °C), molecular weight of the water and density, respectively. *P* is the water vapour pressure and *P_s_* is the water vapour pressure at saturation. *R* is defined as a gas constant and *T* is the absolute temperature [47]. The water condensation occurs in all the pores with radius up to *r_k_*/*2*, at the given temperatures and constant pressures. For a smaller *r_k_* value, or a lower temperature, water vapour condensation occurs in capillaries more easily. The above physical principal mechanisms can be recognized as a basic work principle of the various available humidity sensors and materials.

As respects the diffusivity increase of nanocrystalline metal oxides is dependently influenced by the number and shape of grain boundaries, *ergo* this attribute can be more pronounced in ceramics. One has to take into account in nanocrystalline moisture pervious ceramics the cavity properties such as surface area, size distributions, open pores volume and their structures. To have a desirable processed ceramic involves a least an average grain size, and well-distributed grains with different neck configurations; the volume fraction of pores should be as high as 45%. The highest number and smallest grade of effective pores results in a great rate of vapour uptake, and thus further condensation [93].

**Figure 6.**
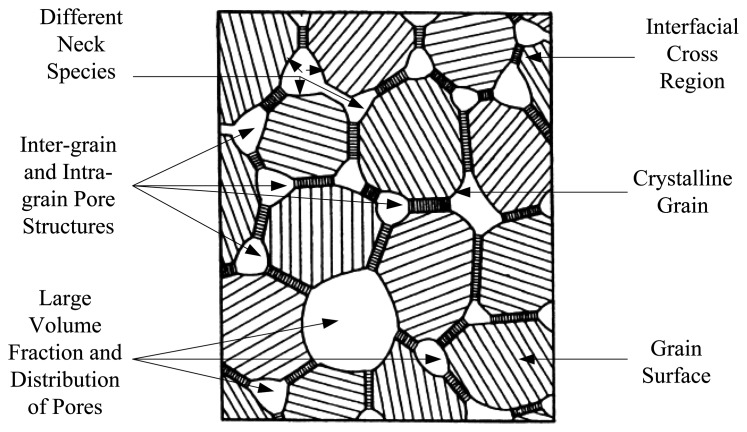
Layout of nanocrystalline ceramic with the different superficial components.

Usually the neck part of the grains exhibits closely similar behaviour to that of bulk materials, therefore, total conductivity of the porous ceramics can be mainly controlled by the neck part of the grains [86]. A schematic sketch of a nanocrystalline ceramic system is depicted in Figure 6. The presence of the crystalline grains with multi-species necks, interfacial cross regions, and widespread volume fraction of pores is visible in the picture [94].

## 5. Impedance Type Humidity Sensors (Resistive)

Resistive type humidity sensors generally contain noble precious metal electrodes either deposited on a glass or ceramic substrate by thick film printing techniques [95] or thin film deposition [96]. The design configuration of most resistive sensors is based on interdigitated (interdigital) electrodes [97] in which the humidity sensitive films are deposited in between them such that they touch the E1 and E2 electrodes. The platform substrate can be coated either with electrolytic conductive polymers such as salts and acids [98,99] or doped ceramic sensing films [100,101]. In some cases, the film-based sensors are formed by applying both printing techniques e.g., screen or inkjet printing, and coating techniques, e.g., chemical vapour deposition (CVD) methods such as spin coating and dip coating, or vacuum physical vapour deposition (PVD) techniques such as thermal evaporation and cold sputtering [102]. In the hybrid structures, frequently the thick film printed layer is the bottom layer. Among the mentioned deposition methods, electrochemical deposition is mostly operative when coating of minuscule area with prepared polymers is required. However, there are rare works in which different deposition methods such as spray techniques [103] or combination of spray pyrolysis with the other techniques [104] were applied.

**Figure 7.**
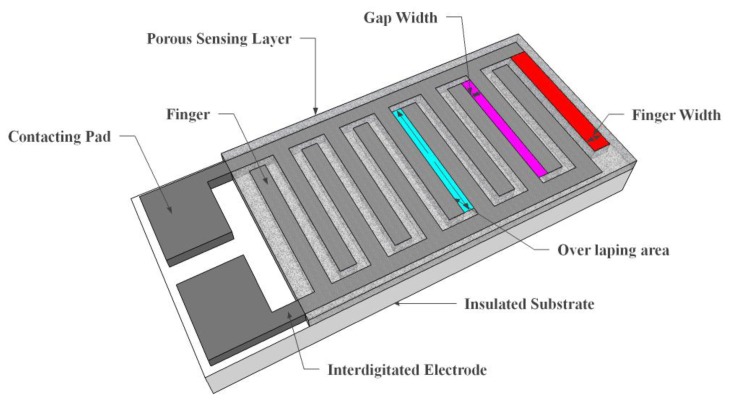
Sketch of a planar thick/thin film-based humidity sensor based on the interdigitated structure with the porous sensing element.

Resistive sensors measure the change of the humidity and translate it into a change in electrical impedance of the hygroscopic medium. Typically, the change of resistance to humidity follows an inverse exponential association, and almost varies from 1 KΩ to 100 MΩ. As a principle, upon adsorption of water vapour its molecules are dissociated to ionic functional hydroxyl groups and this results in an increase of film electrical conductance. Moreover, the response times of resistive sensors mostly range from 10 to 30 s for a 63% change of the humidity level [105]. A prototype sketch of a planar thick/thin film humidity sensor based on the interdigitated structure with the porous membrane is shown in Figure 7. Important parameters of the design are highlighted in the schematic sketch.

**Figure 8.**
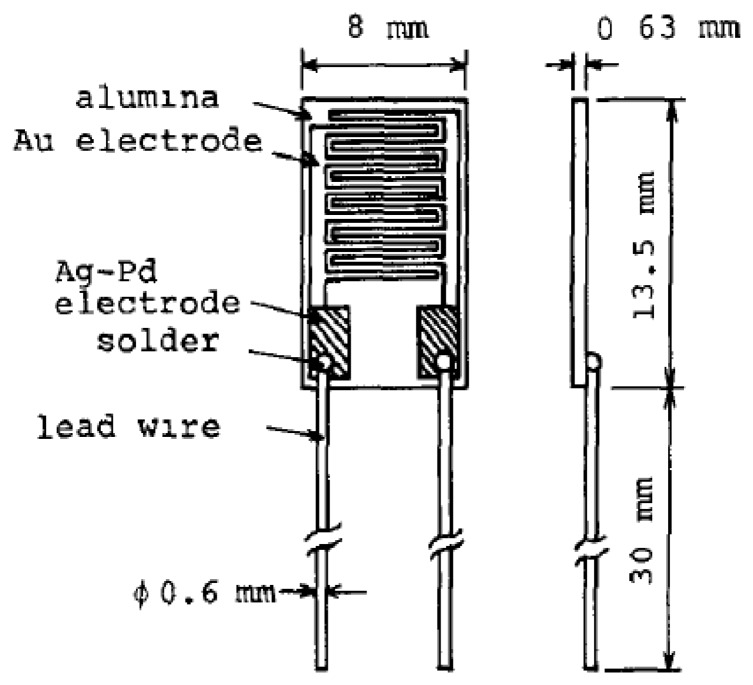
Schematic view of the ‘Hument HPR’ type humidity sensor [47].

An initial thin film resistive-type humidity sensor with a high accuracy of (1%), referred to as ‘Hument’, was developed by the Nakaasa Instrument Co. Ltd. in 1978 [47] and it has entered the market. Figure 8 shows a schematic sketch and the dimensions of this sensor. It was prepared based on the copolymerization of ammonium salts on an Au interdigitated electrode placed upon an alumina substrate. The response time of the adsorption was measured at about 2 min, while it was slightly longer during the desorption process. Hument suffers from long response times and large dimensions. As time passed, other copolymers with different preparation methods such as cross-linked copolymers (networks of cross-linked polymers) prepared from styrenesulphonate by a photo-polymerization technique, vinyl polymer and N,N-methylene-bls-acrylamide as cross-linking regent have been investigated [106,107].

### 5.1. Polymer-Based Resistive Humidity Sensors

Research and studies on polymeric humidity sensors have continued and applied in industry over the last four decades. Most of these sensors are fabricated based on thin films of porous polymers [108], and apply sensing principles similar to those of metal oxide ceramic sensors. The sensors' functionality is based on the physical and chemical water uptake of the films and condensation in the presence of capillary-pores, and therefore a change in some physical and electrical properties of the transducer. The magnitude of the change of bulk conductivity or dielectric permittivity depends on internal confidants.

However, the demand for organic polymer thin film humidity sensors and its applications still has a lower degree of satisfaction and importance compared with that for metal-oxide thick or thin film ceramic sensors [109], but their fabrication and development has progressed continuously, especially in laboratory research works [110]. During the past two decades, resistive elements [105,111] such as thick film planar [112] and thin film evaporated [113] components based on the three groups of polyelectrolyte polymers [114–116] and copolymers [117–119] have been developed for humidity sensing applications [120–124].

Polymer electrolytes (polyelectrolytes) are a group of polymers with electrolytic groups such as ionic monomers which show ionic conductivity when exposure to water vapour. They respond to water vapour variations by increasing their ionic mobility or charge carrier concentration from low to high [125,126]. Polyelectrolytes can be made from salts, acids and bases. According to Chen and Lu's classification, moisture sensitive polyelectrolytes are grouped based on their functional electrolytic groups and can be fundamentally divided into three major categories consisting of quaternary ammonium salts, sulfonate salts and phosphonium salts [49]. Polyelectrolytes are commonly hydrophilic and tend to be soluble in water, and therefore are not resistant to water vapour for a long time and suffer from weak perdurability [47,113]. In addition, polymer electrolytes have a serious deficiency of resistance change when exposed to high levels of humidity, often higher than 50% RH. Having a large hysteresis is also among the other significant disadvantages of these groups. In contrast, conducting polymers are partly hydrophobic and, thus exhibit higher durability due to lower water uptake [126,127]. Besides, conducting polymers like poly(3,4-ethylenedioxythiophene) (PEDOT) or poly(3,4-ethylenedioxythiophene–poly(styrene-sulfonate) (PEDOT-PSS) exhibit higher moisture sensitivity, and significant reversible changes of impedance when exposed to humid conditions [128]. A resistive-type miniature humidity sensor made of an organic conjugated polymer based on PEDOT has been developed and fabricated using standard MEMS technology integrated with a polymerisation process. The electrical test characteristics showed that the sensor is sensitive to a RH range of 20% to 99% [129]. Further, Miyoshi *et al.* reported a flexible design configuration for a resistive humidity sensor based on a sandwiched porous hydrophobic poly-tetrafluoroethylene membrane structure deposited by a soft-MEMS technique applicable to physiological humidity ranges [130].

There have been some improved methods to design and fabricate water-resistant elements in the case of resistive humidity sensors based on polymer electrolytes to reduce the water solubility and protect them from deformation by dissolution [131]. Some of these methods encompass the application of hydrophobic groups by grafting and copolymerization to apply protective films [132–134], cross-linking formation and interpenetrating network structures [135–137], preparation of organic/inorganic hybrid compounds containing metal or metal oxide added polymers [138–140], introduction of reactive functional groups for formation of chemical bonds between substrate and sensing layer by photochemical crosslinking reactions [141–143], anchoring of polymer membranes on the electrode surfaces by means of UV irradiation, mostly in work by Gong *et al.* and colleagues [144–146], and addition of dopants, e.g., salts or acids to fix the shortcomings of semiconducting/conducting polymers [147–149].

Water-durable polyelectrolyte humidity sensors based on composites of epoxy resin and two other comonomers selected from quaternary ammonium salts have been prepared by Lee *et al*. The sensing membrane was formed from composition of glycidyl trimethyl ammonium chloride (GTMAC) as monomer, and methyl tetrahydrophthalic anhydride (MTPHA) and polypropylene glycol diglycidyl ether (PPGDGE) as co-monomers, then injected on a printed gold electrode to form a sensor. The sensors were tested to be used under severe conditions [150]. In the case of polyelectrolyte quaternary phosphonium salts, a new monomer of (vinylbenzyl) tributyl phosphonium chloride was copolymerized with two other co-monomers as a water resistant humidity sensing membrane using a dip coating method [151]. The sensor has shown water vapour sensitivity for a RH range from 20% to 95%. To improve the durability and stability at high humidity, Sakai *et al.* have reported the simultaneous cross-linking and quaternization of poly(4-vinylpyridine) polymer with the other polymer [152]. Later, Sakai *et al.* designed a cross-linked hydrophilic polymer films to form interpenetrated polymer networks (IPN) with a hydrophobic polymer, to make the hydrophilic polymer films more stable and durable in a high humidity ambience [105]. Copolymerization of hydrophilic or hydrophobic monomers with other hydrophobic monomers, and the influence of different parameters on the performance of cross-linked quaternized polyelectrolytes membranes have been further reported in [153,154].

In the past eight years many works have considered ways to address the inherent shortcomings of polymer electrolyte-based sensing membranes, hence eliminating some their undesirable characteristics. By reviewing the published data, it is revealed that most of these concerns were regarding water solubility at high humidity, low degree of sensitivity at low humidity, decrement of overall impedance, and hysteresis modifications. Poly(4-vinylpyridine) is an example of a weak hydrophilic polymer, which was subjected to some treatments to be utilizable as a humidity sensitive material by Li *et al*. To improve the moisture sensitivity, film strength, stability and ability to operate in humid environments, they copolymerized a monomer of butyl methacrylate with 4-vinylpyridine along with reaction of dibromobutane to obtain crosslinked and quaternized structures [155]. Moreover, a novel polyester (PET)-based substrate resistive humidity sensor has been fabricated based on the *in situ* copolymerization of two different polymers to achieve a long term stable sensor [156]. According to Li *et al.* water durable humidity sensing films based on an interpenetrating network (IPN) structure have been simultaneously prepared by cross-linking of poly(dimethylaminoethyl methacrylate) (PDMAEM) and poly(glycidyl methacrylate) (PGMA). 1,4-dibromobutane (DBB) and diethyltriamine (DETA) hydrophobic polymers were used as crosslinking monomers, respectively. The sensor has been tested over 20%–97% RH and it presented low-humidity sensitivity, and an acceptable response of 4 s [157]. Polypyrrole is one of the polymers which has some drawbacks. Sun *et al.* fabricated a polypyrrole composite-based sensor by chemical liquid phase polymerization and further quaternization with 1,4-dibromobutane to obtain a low-moisture sensitive transducer [158]. Other works report a resistive type humidity sensor with a focus on improved preparation methods of crosslinked polyelectrolytes, e.g., quaternization and then copolymerization [159], crosslinking and quaternization [160]. As a newer contribution by Sun *et al.* a polyelectrolyte cross-linked resistive humidity sensor based on copolymerization and then quaternization was proposed and tested in different chemical environments such as acetone and ammonia, at humidity ranges of 30% to 95% RH. As a result, for a 63% change of humidity, the sensor showed an impedance change of four orders. By exposure to ethanol vapour, the output properties only decreased by one order of magnitude [161].

As mentioned earlier, the creation of robust conjunctions between polymers and plastic substrates, frequently polyester (PET) and polyimide substrates, is a main concern of current developments. This challenge has been somewhat mitigated by introducing physical and chemical reactive methods to form a bonding matrix. Humidity sensing composite nanofiber materials of silicon-containing polymer electrolyte, polyethylene oxide and polyaniline have been prepared via an electrospinning method. Both the humidity response and cross-linking cohesion were improved by modification of the first layer (gold electrode) with poly(diallyldimethylammonium chloride) (PDDA) before the deposition. Plus, they found that the presence of polyaniline (PANI) in nanofibers effectively led to a decrease of the film impedance. It was also reported that the short response time of the sensor may be regarded as due to the enhanced junction of the composite nanofibers and substrate [162]. Based on their newer work on polyaniline (PANi)-based nanofiber composites, it was observed that the adhesion of film to both the substrate and electrode was greatly influenced by the formation of nanostructure beads in the nanofibers. Moreover, moderating and maintaining the ratio of poly(vinyl butyral) and poly(ethylene oxide) composition in the electrospinning (ES) solution, resulted in good electrical contact, relatively high specific surface area and modification of the nanofiber's hydrophilicity in bead-containing PANi nanofibers [163]. Anchoring of humidity sensing polyelectrolytes to the electrode surface on plastic substrates is the other alternative. Su *et al.* have fabricated novel flexible resistive humidity sensors based on anchored polyelectrolyte membranes (produced by copolymerization of methyl methacrylate and [3-(methacrylamino)propyl] trimethylammonium chloride) to an interdigitated gold electrode (pretreated with 3-mercaptopropionic acid) on a PET substrate by a peptide chemical protocol. N-(3-dimethylaminopropyl)-N'-ethylcarbodiimide hydrochloride (EDC) was employed as a peptide coupling reagent. The sensors have shown good humidity dependence over wide humidity ranges (20%–90% RH) with good long term stability. The water stability of the anchored poly-MMA-MAPTAC to the MPA/Au contact area was influenced by the amount of added EDC [164]. Improvement of the sticking between polyelectrolyte sensing layers and polyimide films as the flexible substrate has also been studied and experimented on [165] by synthesizing polyelectrolyte and epoxy resins as interpenetrating polymer networks, which were prepared by crosslinking and copolymerization to become a potential method. Sensing membranes were fabricated through a screen printing procedure and evaluated at humidity ranges of 20% to 95% RH with good sensitivity from low to high regions [166]. Most of the reviewed sensors have exhibited humidity sensitivity even at RH values lower than 30%.

To increase the impedance change of those resistive polymeric humidity sensors based on conjugated conducting or semiconducting polymers, doping and dispersing catalyst agent ions inside the precursor materials leads to a decrease of resistivity at low humidity [167–170] and hence greater conductance changes. In this regard poly(*p*-diethynylbenzene) or PDEB have been synthesized with nickel catalyst (Ni-C) as a novel transition metal-acetylide complex catalyst in dioxane toluene mixed-solvent system at 25 °C by Yang *et al.* and are usable for humidity sensing purposes. The result was a compound with higher molecular weight (MW) and good solubility. The sensor was tested in the humidity range of 15%–92% RH, and the impedance of the sensor varied from 10^3^ to 10^7^ Ω, which was enough low compared to other sensors based on conjugated polymers [171]. Gold nanoparticles are among the metal catalysts which have been utilized to provide high-conductivity thin film sensors [172]. Su *et al.* synthesized and fabricated impedance type humidity sensors of amine terminated polyamidoamine (PAMAM) dendrimer polymer dispersed with AuNPs (gold nanoparticles) by drop-coating on a polyester substrate. Their prototype design offers the advantages of flexibility due to the novel substrate, and are low RH sensitive due to the presence of the Au catalyst agents [173].

An additional drawback of polyelectrolytes-based resistive-type humidity sensors, is their poor detection of low humidity when operate in very dry atmospheres (under around 40% RH), where they thus exhibit extremely low conductivity, which consequently makes difficult or impossible output electrical response measurements. To eliminate this problem, one solution is to change the polymer matrix by applying a superconductor with high intrinsic conductivity and mechanical mixing methods. Li *et al.* have developed a poly(4-vinylpyridine)/carbon black (CB) composite to be used as a humidity sensitive material [174]. Based on their work, poly(4-vinylpyridine) (PVP) was grafted onto carbon black (CB) in the presence of TEMPO to form a new compound which was further quaternized and cross-linked with alkyl halides such as bromobutane and 1,4-dibromobutane to obtain a humidity sensing material.

In other work after that, a salt doped (sodium perchlorate-doped) copolymer of quaternary ammonium salt (2-(dimethylamino) ethyl methacrylate (DMAEMA) with poly(ethylene glycol) methyl ether methacrylate (PEGMEMA)) was prepared by Lv *et al.* to be used for resistive type thin film humidity sensors [175]. These sensors were characterized in the humidity range from 10%–98% RH and resulted in a variety of conductance for different ranges of relative humidity, and of note, especially high conductivity was seen even at very low humidity.

### 5.2. Ceramic-Based Resistive Humidity Sensors

Electroceramic materials in both the form of compounds or single-species with single/polycrystalline structures may be envisioned as useful candidates for moisture sensing applications. To resolve the deficiency problems (the problems happen because of deficiency of materials) of conventional materials such as inadequate sensitivity or selectivity, low catalytic grade, insufficiency of cavities, surface degradation due to harsh contaminants in harsh environments and failure to operate in parched or soaked environments, the utilization of the innovative materials with novel humidity detection mechanisms has been propounded [176]. This target can be pursued by formation of newfound combinations *i.e.*, admixing of nanomaterials with different particle size/morphology, hybridization of materials by substitution or doping of the new atoms in the lattice, particle size reduction to sub-nano scales, and finally investigation of the influential properties of materials from different families with different vacancies in the valence layer.

Porous ceramic or nanorod-based ceramic humidity sensors can be realized and formed through techniques such as thick film screen printing [177,178] whereby conductive and nonconductive pastes are deposited onto an insulating substrate, thin films of plasma or vacuum vapour which are deposited based on semiconducting metal oxides [179,180], and films are formed by anodization, mostly for aluminium oxide (Al_2_O_3_) [181]. In such thick film products the film thickness is usually greater than 10 μm and dopant agents can be added to pre-reacted powders as reaction catalysts to promote the dissociation of water molecules into functional groups containing hydrogen and hydroxyl ions. Thin films of vacuum vapour or plasma sputter deposition on various substrates such as silicon will also function as resistive type devices, and mainly utilise ionic-electronic conduction due to their semiconducting nature. The surface hydroxyl ions decrease the film resistivity, and consequently change the impedance.

As mentioned in Section 3, resistive sensors fall into ionic and electronic conduction groups. MgCr_2_O_4_-TiO_2_ [182], ZnCr_2_O_4_-LiZnVO_4_ [183], TiO_2_-K_2_Ti_6_O_13_ [184], Ni(Al, Fe)_2_O_4_-TiO_2_ [185], MgFe_2_O_4_ [69], ZnO,TiO_2_ [186], and nanoscale size SnO_2_ added with different ratios of CuO, Fe_2_O_3_ and SbO_2_ co-oxides [187] are examples of ionic conduction types, which operate by utilising the surface chemisorption and physisorption to measure the ambient relative humidity. A MgCr_2_O_4_-TiO_2_ porous ceramic humidity sensor was developed by Nitta *et al.* for microwave oven applications [188]. This material functioned based on the chemisorption and physisoption of water molecules and finally ionic protonic conduction [86]. The humidity sensing evaluation occurred in two phases comprising low and high RH: at low RH, chemisorption caused dissociation of water vapour molecules and establishment of hydroxyl ions at the surface of the Cr^3+^ sites. Increased humidity levels cause physisorption and formation of the first layer of hydronium ions and therefore proton transfer to the neighbouring sites and this leads to conductivity enhancements (ionic or protonic conduction). At higher RH, the water vapour condensation occurs in the capillary-quasi apertures to form liquid-like layers. The electrolytic conduction leads to the occurrence of further conductivity. As a major conduction mechanism for most of the available humidity sensors, this type of transduction has been previously detailed in Section 4.

**Figure 9.**
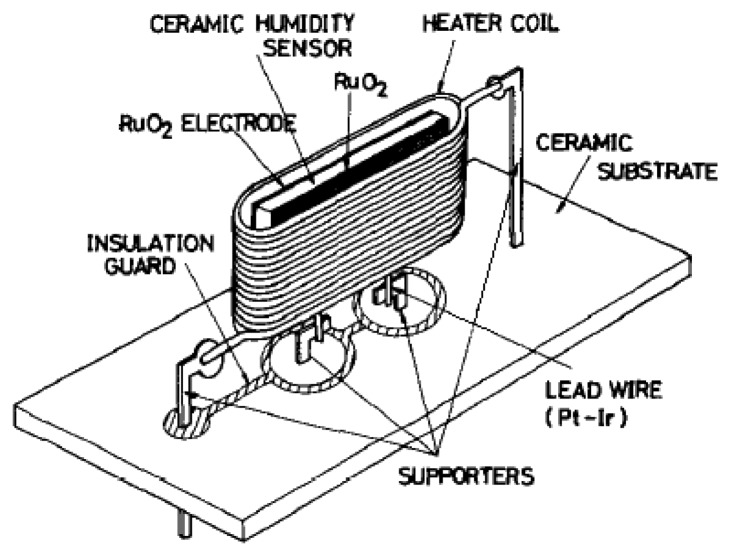
Construction sketch of MgCr_2_O_4_-TiO_2_ ceramic humidity sensor [86].

The porosity, average grain size and average pore size of MgCr_2_O_4_-TiO_2_ were 30%–40%, 1–2 μm and 300 nm, respectively. A heater enhanced the humidity sensor sensitivity by heating up it to 450 °C after any time of usage. This heating was necessary to eliminate the hydroxyl groups on the surface. Moreover, the heater removed contaminants such as dust, oil and other chemical vapours. Figure 9 shows the construction structure of this sensor.

Yamamoto *et al.* studied and proposed a similar sensing mechanism for a TiO_2_/SnO_2_ (mol ratio 1:1) moisture sensor based on the ionic conduction mechanism on the sensor surface [189]. In this case, the grains with the larger cross section of intergranular necks, made the device less sensitive to chemisorption, and hence lessened the effects of hydroxyl functional groups. Plus, the use of this ceramic sensor without a heater element would be possible in an environment with no toxic gases or harmful dust. Around eight years later on 1989, a porous ceramic humidity sensor made of pure titania was investigated by Yeh *et al.* [190]. As a novel finding of this work, the sensor could be operated at room temperature (25 °C) and recovered without any need of thermal desorption. Furthermore, the humidity-conductance sensitivity of the sensor was changed by more than four orders of magnitude for a RH range of ∼15% to 95% at 400 Hz. Based on the outcome of the electrical characterization, the sensor was operated by means of ionic conduction carriers which were both ions and electrons, and ions were the dominant carriers.

The humidity sensing properties of magnesium ferrite (MgFe_2_O_4_) and magnesium ferrite substituted with different ratios of lithium (Mg_1−x_Li_x_Fe_2_O_4_) (0.2 ≤ x ≤ 0.6), synthesised from inorganic precursors through a solid state reaction have been investigated [191]. The purpose of Li^+^ ion substitution was mentioned to be a further decrease of the spinel ferrite ceramic grain size, therefore porosity promotion and creation of more lattice defect structures. The humidity response of the composites was evaluated in a RH range of 10%–80% at room temperature. All the lithium ion-substituted spinel ferrites have shown increased humidity sensitivity, especially the x = 0.2 Li substituted sample. At low RH the sensitivity of the alkali-contained samples increased due to better electron donation on the bulk surface from the water vapours. Overall, a sample with x = 0.2 Li provided a high surface area and high degree of porosity, hence stronger adsorption/desorption. By data comparison it has found that the lowest porosity and thus least sensitivity corresponded to the x = 0.6 Li sample.

Humidity sensors based on semiconducting metal oxides were subdivided as electronic conduction type sensors, and were mainly studied at temperatures higher than 100 °C. Since these sensors operate at temperatures much greater than that of water physisorption, therefore, their conduction mechanism is only based on the chemical adsorption (chemisorption) of water vapour molecules [48]. In fact this type worked based upon the electron donation from water vapour molecules to the semiconductor metal oxide surface. In such cases the water vapour chemisorption would result in either an increase or decrease of sensor resistivity correlating to the semiconductor type, whether is n type or p type. Microstructure properties of semiconducting electronic type sensors, such as surface area, pore size distribution and average particle size are not as effective as those ionic types microstructures for the sensing characteristics of the sensor [47]. Based on Yamazoe and Shimizu's report, (Sr_1−x_, La_x_) SnO_3_ and ZrO_2_-MgO are among the electronic types, however this is the only work in which the perovskites such as SLS were placed in the high temperature semiconductor group of sensors. Maybe the substitution of the B and X sites with lanthanum and tin oxides affected the working principle of the sensors. In other work in the early 1980s Seiyama *et al.* declared that the conductivity change of the n-type semiconductor electronic sensors *i.e.*, SrSnO_3_, is larger than that of p-type sensors such as SrTiO_3_, when exposed to humidity [69].

#### 5.2.1. Perovskite Type Ceramic Humidity Sensors

Research and study of the humidity sensing behaviour and morphological structure of porous perovskite films and bulk materials continues to give rise to novel tentative and innovative results [192–194]. Perovskite films have been applied to produce capacitive humidity sensor transducers [195] and micro devices [196]. The humidity sensing mechanism of perovskite type oxides with general formula of ABX_3_ was reported to be based on the electron donation from water vapour molecules. It was claimed that their activity can be attributed to the trapped electrons due to the presence of surface defects such as ionized oxygen vacancies, and these trapped electrons may be liberated by adsorption of water vapour molecules on the defect sites [48,69]. Based on the previous researches, it has been reported that the humidity-sensitive properties of perovskite ceramic oxides are only exhibited at elevated temperatures ranging from 300° to 500 °C, thus, they operate based upon the electronic conduction type mechanism [48,69,197]. For cerium-based perovskites such as Nd_2_O_3_ doped BaCeO_3_ and SrCeO_3_ it has been found that the mobility of protons is higher than the mobility of oxygen ions at the temperature of 700 °C, therefore they are used for potentiometric humidity sensor applications due to their high proton conductivity [198,199]. A solid electrolyte perovskite-based humidity sensor of SrCe_0.95_Yb_0.05_O_3_ for high temperature applications responded to humidity by mixed conductors of hydrogen ions (protons), oxygen ions (electrons) and positive holes as lattice defects [200]. In other work mixed perovskites of barium calcium niobates (BCN) have shown good promising proton conductor properties, and stability in low temperature humid atmospheres. In this material proton mobility is about one order of magnitude of the oxygen ion concentration and changes of conductivity are a function of the H_2_O dissociation, hence it could be a ionic conduction type complex [201].

From around 1987, it was specified that many kinds of perovskite metal oxides with porous structures demonstrate robust humidity-sensitivity characteristics even at room temperature due to their protonic ionic conduction sensing mechanism and lack of electronic conduction [202,203]. Depending on the applied precursor for A or B sites of the complexes these sensors showed humidity sensitivity from 8%–20% to higher RH values of around 98% [204–207]. Sadaoka *et al.* revealed that KH_2_PO_4_ doped porous PLZT (Pb,La)(Zr,Ti)O_3_ ferroelectrics (potassium dihydrogen phosphate-doped lead lanthanum zirconate titanate) sintered at 700 °C can be used as a humidity sensing element at room temperature and a humidity range of 0 to 90% RH. Furthermore, they observed that humidity dependence of the impedance of the sensor was mainly based on the coverage of adsorbed water vapour (physisorption), and therefore dominant carriers are protons with ionic conduction [208].

Yeh *et al.* [209] attempted to study the humidity sensing physical and electrical characteristics of porous Ba_0.5_Sr_0.5_TiO_3_ ceramics in different low to high frequency ranges at room temperature (25 °C), and indicated that this perovskite is a suitable candidate to be used as humidity sensor material. They found that the dominant conductive carriers are probably protons, thus the sensing principle is protonic ionic conduction. This composition had shown good sensitivity at low frequency in a normal ambience with no toxic gases, and was reversible between 15% and 95% RH at low temperature. In other work [210] they have investigated the effect of dopants such as potassium oxide (K_2_O) on Ba_0.5_Sr_0.5_TiO_3_ sintered at 1250 °C. The new composition's conductance changed by as much as four orders of magnitude at low frequency for the same RH range, while the undoped one was changed by around three orders of magnitude. The microstructures of the doped specimens such as open porosity and specific surface area have shown a slight decrease compared to those of pure BS samples.

In these cases, perovskite type sensors are mainly operating based on electrical properties which encompass the resistance and capacitance changes associated with the ambient relative humidity variations, hence could be regarded as impedance or capacitive ceramic sensors [211–213]. As far as ionic conduction is strongly dependent on the formation of monolayer and multilayer by physisorption and capillary condensation of the water vapour molecules, therefore perovskites' humidity sensing characteristics largely depend on the film or bulk complex microstructures, such as specific surface area, effective porosity, volume and pore size distribution. Accordingly, microstructure control of the porous elements is highly determinative of sensor efficiency [214]. Chang *et al.* have presented that the humidity-conductance and sensitivity characteristics of calcium titanate perovskite sintered at different temperatures is strongly dependent on the microstructure of the sintered ceramics [214]. Meanwhile, it was reported that the moisture sensitivity of these structures depends on the interfacial microstructure and material compositions. As a test result, Sr(Sn_0.5_,Ti_0.5_)O_3_ has found to display quick response, and worked independently of the ambient temperature. Further, the ceramics in this research have been prepared by skipping the calcination process, and they were directly sintered. The obtained ceramics had shown higher porosity than those that were subjected to calcination processes [215]. A spin-coated humidity sensor of a novel bismuth potassium titanate (Bi_0.5_K_0.5_TiO_3_) perovskite on a silver-palladium intedigitated electrode and alumina substrate has been fabricated by Zhang *et al.* [216]. BKT sensing powder was synthesized via chemical solution chemistry. In this case a sensing paste was directly prepared by mixing powder and deionized water without any binder or annealing stage. To investigate the sensing mechanism of the sensor it has been subjected to morphological and qualitative phase characterizations and further humidity sensing investigation in the range of 11% to 95% RH. From the results it was claimed that the sensor is utilizing surface interactions and the conductivity changes are mainly due to the ion charge carriers by hopping transport because of the high local charge density. The sensor has shown an impedance change of around four orders of magnitude for the entire range of relative humidity tested, and hysteresis of about 3% RH.

The barium perovskites family, e.g., BaTiO_3_ [217–220] or composites of BaTiO_3_ and polymers [221–223] with porous structure and various operating temperature have attracted considerable attraction for moisture sensing applications. Viviani *et al.* have studied the humidity sensing behaviour of porous barium molybdenum oxide (BaMO_3_) with compositions of M = Ti, Zr, Hf, or Sn prepared by wet chemical synthesis and different elevated temperatures ranging from 1,250 °C to 1,700 °C. The samples with lower sintering temperature and higher open porosities have shown an increase of both conductance and capacitance with an increment of the humidity range from 20% to 80% RH at room temperature (25 °C). They reported that highest sensitivity was found in highly porous BaTiO_3_ specimens doped with the addition of 0.3 at.% of La. Based on the experimental results they claimed the sensor is operating according to the surface protonic conduction model [224]. In other research by Hwang *et al.* the electrical characteristics of porous lanthanum (La) doped BaTiO_3_ have been studied by means of complex impedance spectroscopy as a function of different sintered densities and humidity conditions. It was revealed that the samples with lower density and higher resistivity exhibited large and nearly linear conductivity in response to humidity changes [225]. Wang *et al.* investigated the humidity sensor resistance properties of sensors made of nano-crystalline BaTiO_3_ doped with alkali carbonate oxides of Li_2_CO_3_, Na_2_CO_3_ and K_2_CO_3_ at 3 wt%, and nano-crystalline BaTiO_3_ sensors without any doping. They declared that the humidity sensor resistance can be decreased to the range 1–10^3^ kΩ by addition of 3 to 5 wt% K_2_CO_3_ or Na_2_CO_3_. Furthermore, they found the addition of NaH_2_PO_4_ to BaTiO_3_ could be the cause of a reduction of the humidity sensor hysteresis [226].

Recently, one-dimensional perovskite-based nanofiber humidity sensors prepared by electrospinning techniques have gained considerable importance due to the higher efficiencies caused by the larger contact areas of surface fibres and moist air. Various kinds of the perovskite materials such as nanofiber barium titanate or barium strontium titanate have been studied for use in humidity sensing applications [227–229]. To reduce the hysteresis loop, BaTiO_3_ nanofibers were synthesized by an electrospinning technique as humidity sensing materials by Wanga *et al*. DC humidity sensors have been fabricated by deposition of barium titanate nanofiber pastes (mixtures of powder and deionized water) on a glass substrate by dip coating on two different as-deposited Al and Ag electrodes. By testing the sensors in a RH range of 11% to 95%, that one based on the silver electrode has shown higher sensitivity and faster response/recovery time, which could be due to the lower resistivity of the metal conductor. Overall results demonstrated the excellent potential of the BaTiO_3_ nanofibers for humidity sensing applications [230]. Recently, electrospun humidity sensors of cadmium titanate (CdTiO_3_) based on one-dimensional nanofibers with porous structures, have been proposed by Imran *et al*. The nanofiber sensor was consisted of different structures with three layers. In this design the CdTiO_3_ nanofibers were deposited using a photolithography technique on a glass substrate, and two aluminium electrodes were placed on top of that with a 25 μm gap width. The electrical humidity sensing characteristics of the sensor were measured experimentally in a 40%–90% RH range at 25 °C by varying the frequency. The performance characteristics of the sensing device such as sensitivity and hysteresis were remarkably improved. Fast response and recovery times of 4 s and 6 s were measured, respectively. The presence of the porous nanostructure, and nanofibers with tiny diameter in the sensing film are among the optimization parameters studied [231].

In many perovskite structures, group II elements (alkaline earth metals) such as Be, Mg, Ca, Sr, Ba and Ra can serve as metal ions to be used as hygroscopic materials to improve the humidity sensing performance. In this regard, due to conduction mechanism of the most perovskite complexes upon interaction with water, they are well-known to the positive ionic conductors at room temperature. Most perovskite oxides are synthesized by chemical solution methods involving mixing and stirring, or solid state reactions by bulk calcination of mixtures of two or more carbonates and/or metal oxides [232].

#### 5.2.2. Thick Film Ceramic Humidity Sensors

Thick film technology (TFT) has been very popular for miniaturizing various kinds of the sensors to be integrated in monolithic hybrid circuits [233,234]. Thick-film technology represents a number of specifications those are very relevant to the understanding of sensors. The advantages of thick film technology for the development of material science, sensor roadmaps, miniaturization and robust products are discussed in [235–239]. Incorporation of nano-scale mixed metal oxides for configuration of thick film sensing layer is one of the principal aims of today's developments, which are being further reviewed.

Thick film humidity sensors based on ZnCr_2_O_4_-TiO_2_ (ZCT) ceramic materials have been conceived and produced by Golonka *et al.* [240]. Furthermore, they studied of different influence of dopants such as Li_2_O, B_2_O_3_ and Si, as well as the effects of sintering temperature on the films' characteristics by equivalent circuit studies using impedance spectroscopy. A thick film humidity sensor using the new ZCT was as good as the MCT (MgCr_2_O_4_-TiO_2_) sensor made by Nitta *et al.* [188]. Li_2_O additions significantly decreased the resistance of ZCT sensor.

Around the mid-1990s, a *p*-type semiconductor MnWO_4_ doped with LiCl received great attention for use as a thick film ceramic sensing element [241–243]. A thick film of porous MnWO_4_ material integrated with a polarity reversed interdigitated platinum electrode prepared by film sintering on an alumina substrate was reported by Qu and had shown good linear response to over 30% RH with a short response time of 3 s and a recovery time of around 15 s at room temperature [244]. Lithium chloride (LiCl) was used as an adhesion promoter instead of the more traditional glass frits for sintering of MnWO_4_, and further allowed the film to be sintered at a lower temperature of 850 °C instead of the 1300 °C used for pure MnWO_4_ film. Consequently, the amount of LiCl influenced the response curve, and the response curve with 5% LiCl was different from the others. They reported that the higher amount of dopants introduced more Li^+^ ions, thus resulting in smaller resistance for lower humidity ranges. SEM micrographs of the sensor showed different grain boundaries and pore size distributions of the material in correlation with the concentration of the LiCl dopants [242]. According to the SEM images, the large amount of LiCl increased the density of the ceramics, and resulted in higher resistance at high humidity levels due to poor water vapour adsorption. Based on their other work [245] on morphology of the thin and thick film manganese tungsten oxide sensors, notwithstanding the short response/recovery times, thin films were less sensitive than thick films regarding the lack of capillary pore structures.

A two-layer ceramic thick film humidity sensor of MnZn ferrite based on an interdigitated electrode structure has been fabricated and presented by Arshaka *et al.* [246]. The sensors were tested at a relative humidity range of 30% to 95% RH and they proposed that the sensor is utilizing a combination of either protonic conduction (ionic), and electron release (electron donation) to the conduction band by oxygen vacancies. Sensors were sintered under two different conditions of air and vacuum. The air-fired sensor shown the highest sensitivity of 1.54%/RH% compared to that prepared under vacuum conditions with a sensitivity of 0.043%/RH%. In other work by these authors with the similar materials, it has been confirmed that the dominant mechanism of conduction is tunnelling [247]. The humidity conduction mechanism of thick film porous TiO_2_ anatasa layers (nanoparticles of ∼16 nm from XRD) fabricated by screen printing on a glass substrate covered by a SnO_2_ (∼200 nm thickness) conducting layer, has been analyzed by AC characterization (impedance spectroscopy) from 2.3% to 60% RH at 24 °C. Based on the study the impedance contained two parts that have both shown humidity dependence: at a low frequency range the electrode polarization responds while at high-frequencies the ionic conduction (proton hopping) is dominant. Further, the humidity variation of the conductivity at low frequency can be considered as the fundamental percolation theory of conduction through percolation clusters formed by adsorbed H_2_O molecules [248]. Resistive-type screen-printed thick film humidity sensors based on synthesised BaTiO_3_ and an interdigitated structure have been fabricated and studied by Wagiran *et al.* [249]. The sensors were tested in a humidity range of 20%–95% RH and showed good linearity and small hysteresis with response and recovery times of 7 s and 15 s, respectively. Sensor resistance was changed by three orders of magnitude from 10^7^ to 10^4^ Ω and the conduction mechanism was identified to be ionic inside capillaries in which protons were the charge carriers.

Thick film humidity sensors based on nano-zirconium oxide (ZrO_2_) on silicon substrate and grain size of 20 nm have been introduced by Wang *et al.* [250]. Sensors were tested in a RH range from 11% to 98% and the impedance was changed by four orders of magnitude from 10^6^ to 10^2^ Ω. They reported that the conduction mechanism in low RH ranges was mainly dominated by polarization of the grains, while for a higher range of RH, the process occurred by dissociation and polarization of water molecules. To be incorporated as sensing element in a screen printed thick film humidity sensor, a nanoparticle mixed-oxide ceramic of Gd_0.2_Ce_0.8_O_2−δ_ (GCO) was synthesized by Hao and co-workers [251]. A sensor consisted of GCO as a top layer printed on a pair of interdigitated electrodes onto a ceramic substrate. The sensor was characterized in the range of 10%–98% RH and different frequency ranges from 20 Hz to 100 kHz. As a result, the impedance was changed by five orders of magnitude at 30% to 98% RH, and acceptable logarithmic linearity was observed in the mentioned RH range in the low frequency region (20 Hz–1 KHz). Further, the sensor showed remarkably low response and recovery times of 40 s and 210 s, respectively, which can be due to the sensor structure design. Based on a significant change of the GCO conductivity at high RH due to hydrogen transport, its sensing mechanism was reported to be a superior ionic type.

The humidity sensing properties of mesoporous ZnO-SiO_2_ composites synthesized by sol-gel methods and fabricated through screen printed films with different Si/Zn molar ratios have been investigated by Yuan and co-workers [252]. By evaluation of the sensors within the range of 11% to 95% RH, it was revealed that introducing ZnO improved the humidity sensitivity of the composites and the specimen with a Si/Zn ratio of 1:1 showed the most promising results among the others. The sensors' impedance changed by more than four orders of magnitude over the whole RH range and low hysteresis also observed. The sensor has also shown slightly high response and recovery times of about 50 s and 100 s, respectively. The analysis of the XPS data suggested that Si–O–Zn bonds existed in the ZnO-SiO_2_ composites and the sensor was utilizing the protonic conduction mechanism. The authors reported the water vapour sensitivity of ZnO-SiO_2_ (1:1) composite was as well as Li-doped mesoporous SBA-15 [253]. The benefit of using ZnO instead of LiCl as a composite part is the reduction or elimination of the water solubility and thus longer stability is achieved. LiCl is a hydrophilic material and long exposure to humidity can caused degradation of its properties [254]. Later, an impedance type thick film humidity sensor of mesoporous iron oxide/silica (Fe_2_O_3_/SiO_2_) composites (Fe/Si with molar ratio of *r* = 0.5) was fabricated for the first time by Yuan *et al.* [255]. The sensing elements were synthesized through a hydrothermal route. This sensor exhibited faster response and recovery times of 20 s and 40 s, respectively, along with excellent linearity. Based on the complex impedance analysis, the sensing mechanism was assessed to be due to hydrogen transport (proton hopping) at low to high RH. Further, in this study the excellent performance of a humidity sensor was reported to be attributable to the microstructure properties resulting from N_2_ gas sorption and HRTEM such as high BET surface area with large volume of pores, and a highly ordered porous structure.

Gusmano *et al.* indicated that the electrical responses of MgFe_2_O_4_ screen printed thick film humidity sensors are in good correlation with their microstructures [256]. The sensors were characterized by means of EDS, SEM and electrochemical impedance spectroscopy (EIS) techniques and the material was found to be a suitable candidate for moisture sensing matter. As shown by DC measurements, MgFe_2_O_4_ thick film sensor conduction occurred through electron carriers at low humidity, when coverage of the oxide surface with water molecules was not completed. At high RH values, both ions and electrons were responsible for the conduction and ions are dominant carriers. Other thick film humidity sensor devices were constructed and developed based on the surface adsorption and capillary condensation such as titania thick film fabricated through low speed spin coating technique [257], or nanocrystalline zinc tungsten based thick films [258].

#### 5.2.3. Catalyst-Added Ceramic Humidity Sensors

There are many works which describe the properties of susceptible ceramics in the form of both bulk processed sensors [259–262] or thick film humidity sensors [263–266] made from various compositions containing multiphase additives and dopants. The introduction of small amounts of dopant ions has a desirable influence on ceramic humidity sensors. Yamazoe suggested that alkali or other dopant ions can act as dominant charge carriers instead of protons, and thus lead to higher humidity-sensitive devices [47]. The addition of 1 mol% MgO or CrO_1.5_ and FeO_1.5_ to ZrO_2_-TiO_2_ increased its conductivity and sensitivity across the humidity range of 20%–90% RH [267]. In another case the addition of 2 mol% K^+^, Li^+^ or Na^+^ to poorly sensitive MgFe_2_O_4_ [47] and 1 mol% K^+^ to NiWO_4_, ZnWO_4_ and MgWO_4_ [268] resulted in their humidity-sensitivity improvement. In case of ZnCr_2_O_4_ LiZnVO_4_, the hydrated Li^+^ ions seem to contribute to the charge transport and enhanced humidity sensitivity [47]. The influence of 0, 1, 2, 5 and 10 mol% of Li, Na and K alkaline earth metal oxides on the humidity-sensitivity response of Nb_2_O_5_-doped TiO_2_ was investigated by Katayama *et al.* [269]. Dopant admixtures affected moisture sensitivity as well as the surface microstructure, however among the dopants, K_2_O with a pore size of less than 0.01 μm greatly influenced the sensor characteristics such as sensitivity and response time.

The addition of alkaline oxides of Na_2_O and K_2_O (5, 10, 20 wt.%) to MgCr_2_O_4_-TiO_2_, help to reduce its pore size distribution, therefore improving its humidity-sensing characteristics at different RH levels [270]. Joanni *et al.* have investigated the addition of Li_2_O to ZnO ceramic sensors, which resulted in improved sensitivity and long time stability as well as decreased resistivity and hysteresis. They indicated that Li_2_O dopants can play the role of sintering agents or liquid glassy phases [271]. In addition, the Li_2_O additives increased the effect of the electrode materials on the electrical properties when exposed to different humidity levels [272]. In a newer work, the DC electrical and humidity sensing properties of mixtures of chromium(III) oxide (Cr_2_O_3_) and tungsten(VI) oxide (WO_3_) with different mole ratios were investigated. Based on the structural and gas sorption studies, composites were found to have a pore radius from 1 to 4.5 nm, which indicates the presence of the micro- and meso-porosity and suitability for humidity sensing applications. By examination of the sensors in the range of 5%–98% RH at room temperature (25 °C), the COWO-14 sample (CO:WO with the mole ratio of 1:4) came up with the highest humidity sensitivity factor [273].

The effects of alkaline earth metal ions from group II have also been emphasized and experimented on the ceramic-based sensors. Humidity sensing, morphological, structural and gas sorption properties of strontium(II) doped (in molar ratios of 0, 0.2, 0.4, 0.6, 0.8, 1) spinel composites *i.e.*, cobalt alumina (CoAl_2_O_4_), zinc alumina (ZnAl_2_O_4_) and barium alumina (BaAl_2_O_4_) have been studied by Vijaya and co-workers. All the composites were prepared thorough the sol gel technique and formed by conventional bulk processing. Similarly, all the specimens were fired under the same conditions of 900 °C for five hours and the DC electrical properties characterized at relative humidities in the range of 5% to 98% RH. As a general result of all the experiments, addition of Sr as well as increases of its molar ratio have led to enhancements of the sensitivity factor. The greatest sensitivity was observed for the composites consisting of 0.8 mol% strontium in CoAl_2_O_4_, ZnAl_2_O_4_ and BaAl_2_O_4_, while the undoped composites possessed the lowest sensitivity. In all three experiments, the mentioned highly sensitive specimens have shown response and recovery times between around 120 s and 50 s [274–276]. The humidity sensitive properties of low temperature sintered pellets of silver-loaded tungsten oxide (0–4 wt% Ag added) have been investigated in the 20% to 90% RH range. The void concentration and sensitivity factor were found to be enhanced with increased Ag concentration. Thus a 4% Ag-doped WO_3_ sensing element showed the maximum sensitivity (2.38 MΩ/%RH), while the pure WO_3_ sample revealed the least (2.11 MΩ/%RH). Based on the results, the mechanism of conduction was announced to be electronic type and water chemisorption on the lattice sites of WO_3_ in a two steps mechanism [277]. Recently, research on the humidity sensitive behaviour of lithium doped maghemite (Li doped γ-Fe_2_O_3_) nanopowders has indicated that the addition of Li is beneficial for lowering the impedance at low RH regions. Moreover, addition of Li with a concentration of 0.45 mol% showed an optimum performance with more linear response. The sensors based on these composites utilized the protonic type operation mechanism [278].

According to recently developed thick film humidity sensors, the influences of alkali metal ions have also been surveyed in thick film-based sensors. Addition of Na_2_O to humidity sensitive TiO_2_-Cu_2_O screen printed thick films has led to decreased sintering temperatures and further grain growth. Desirable microstructure features of films for moisture sensing applications such as large surface area and porosity have been achieved by addition of amounts of sodium oxide. The sensors showed resistance change of three orders of magnitude between 20% and 95%RH as a common humidity sensor [279]. Songa *et al.* introduced a fast, simple and reliable thick film screen printed humidity sensor of potassium chloride (KCl) doped SnO_2_ nanofibers based on an interdigitated structure. The sensor showed very low hysteresis, fast and very close response/recovery times of 5 s and 6 s, respectively, for a range of 11% to 95% RH. They claimed that the sensors' impedance was changed by more than five orders of magnitude for this RH range [280].

#### 5.2.4. Doped and Undoped Semiconducting Thin Film Humidity Sensors

Thin film humidity sensors based on ceramics exhibit favourable microstructure specifics as well as higher chemical resistance and mechanical strength than polymeric sensors. Some deficiencies in case of the films sputtered with PVD techniques such as difficulty in control of the film microstructure which may be further deteriorated and caused to the presence of the pinholes, have led to the consideration of CVD deposition techniques with interest in the use of the sol-gel method for production of ceramic humidity sensors [281]. This method is considered a cost effective processing route which allows the synthesis of high purity nanostructure ceramic thin films with controlled homogeneous microstructures, and further decreases the total size of the sensors. The method was also applied for preparation of amorphous glassy thin film systems [282,283].

Thin film TiO_2_-K_2_O-LiZnVO_4_ ceramic humidity sensors have been prepared by the sol-gel method with different molar ratios of precursors. Addition of potassium alkali and glass phases to the rutile phase of TiO_2_ has led to excellent linear responses of resistance and capacitance curves over the 10% to 99% RH range at low frequency values [284]. Thin film humidity sensors of In_2_O_3_/SiO with different SiO compositions (from 5% to 45%) have been developed by Arshak *et al.* using low to high vacuum pressure. The results indicated that 85% In_2_O_3_/15% SiO fabricated at a vacuum pressure of 2 × 10^−4^ mbar is promising for use as a humidity sensor [285]. Elsewhere the authors pointed out that the addition of 20 wt.% TiO_2_ to SnO_2_ thin films showed the highest sensitivity and three orders of magnitude change for 20% to 90% RH [286].

Moreover, in the field of thin film-based ceramic humidity sensors many works have been published based on the use of nanosize zinc oxide or ZnO doped with other alloys and chemicals [287]. Spin-coated nanorod thin film humidity sensors of aluminium doped zinc oxide have been prepared by Sin *et al*. The sensing elements were prepared through the sol-gel technique. By increasing the dopant concentration, the length of the nanorods and current increased, therefore a sensor that contained 0.6 at% aluminium-doped ZnO exhibited the highest sensitivity over the humidity range of 40%–90% RH [288]. Spin coated humidity sensors based on ZnO thin films were fabricated on a silicon wafer substrate by Hsu and co-workers, and grown by means of a vapour solid (VS) method at various temperatures from 400 to 700 °C. The results indicated that the films grown at a temperature of 700 °C were the most sensitive specimens. The mentioned sensors showed a resistance change exceeding 10^4^ times in the 11% to 95% RH range at room temperature [289].

There are further improved works which realized these types of humidity sensors based on sputtering techniques. A thin film humidity sensor of nanostructured zinc oxide with a polycrystalline phase was produced by DC reactive magnetron sputtering. The film's impedance spectroscopy results indicated lattice defects, therefore oxygen vacancies promoted the adsorption rate of water vapour. By characterization of the sensors in the relative humidity range of 6.3% to 84%, it was seen that the resistance was changed by nearly four orders of magnitude, and the device showed a very fast response and recovery time of 3 s and 12 s, respectively [290]. Micro ZnO-In_2_O_3_ thin film humidity sensors were produced by radio-frequency sputtering based on an interdigitated electrode structure (layer by layer sputtering of ZnO and In_2_O_3_ precursors with the same deposition time and different number of layers). The specimen produced by applying ZnO two times and In_2_O_3_ one time showed the best characteristics as determined by the total impedance changes of more than four orders over the RH from 11% to 95%. This was explained by the fact that the improved humidity sensing behaviour was due to the heterojunctions between the ZnO and In_2_O_3_ films. The sensor response and recovery times were about 15 s and 40 s, respectively [291].

A nanoporous thin film of TiO_2_-10 mol% SnO_2_ co-doped with 0.3 mol% La^3+^ and 50 mol% alkali metal (K^+^) has been reported by Anbia *et al*. They found that the addition of La^3+^ rare earth metal ions, K^+^ ions and Pluronic copolymer improved the humidity-sensitivity of the samples by an impedance change of 10^9^ to 10 ^4^ Ω and showed excellent linearity in room temperature [292,293]. In other work 0.5 wt% of La^3+^/Ce^3+^ co-dopants on TiO_2_-20 wt.% SnO_2_ thin film sintered at a low temperature of 500 °C, led to a five orders of magnitude impedance decrease from 10^9^ to 10^4^ Ω for a humidity range of 15% to 95% RH [294].

Nano-structured ZnSnO_3_ has been synthesized by a chemical precipitation method using AR grade chemicals of ZnO and SnO_2_. For optical humidity sensing studies of the materials, the sol-gel spin coating technique was used for deposition of a uniform thin film of chemical solution. Based on XRD patterns, ZnSnO_3_ was found to have a perovskite crystalline phase and orthorhombic structure with minimum crystallite sizes of 4 nm. Results have shown that annealed ZnSnO_3_ pellets with 1:4 weight ratio were the most sensitive specimens (maximum sensitivity of 3 GΩ/RH%) *versus* humidity compared to pure SnO_2_ with the same characterization conditions [295].

Recently, a humidity sensitive SnO_2_-SiO_2_ mesoporous composite was synthesized via a one-pot sol-gel method. Thin film humidity sensors were fabricated by dipping quartz crystal microbalances (QCMs) into the coating solutions. By exposing the QCMs coated with SnO_2_-SiO_2_ thin films to relative humidity within the range of 11% to 96.1% RH at room temperature, the sensor with a Sn/Si atomic ratio of 1:1 revealed better moisture sensing behaviour, including suitable sensitivity and low hysteresis. The sensors response and recovery times were found to be about 14 s and 16 s, respectively [296].

#### 5.2.5. Doping Influence on Perovskite's Humidity Sensing Properties

ABX_3_ is a general structure of perovskite-type oxide materials. A minor change to the common structure in the A or B metal sites may contribute to an improved humidity sensing response of the resulting compound at different RH ranges with different components. There are some recent works in which the influence of additional ions or substitution of various oxides in different sites of the perovskite ceramics of humidity sensing performance have been investigated [297,298]. Based on newly developed thick film humidity sensors, a number of works have recently been focused on the composite synthesis for fabrication of doped thick film products [299,300].

Humidity-conductance characterization of K_2_O doped (Ba_0.5_,Sr_0.5_)TiO_3_ has shown that the conductivity change of the doped specimens is one order greater than that of undoped materials [301]. The humidity sensing characteristics of undoped, CaO and MgO doped (Ba_0.5_,Sr_0.5_)TiO_3_ in the bulk form and of thick solid state sensors have been investigated by Slunecko *et al.* [302,303]. As a result for bulk ceramics, the humidity-conductance response of the doped samples were changed four orders of magnitude, while three orders of magnitude changes were measured for undoped samples in the humidity range of 10%–90% RH and frequency of 400 Hz. The microstructures of the BST materials such as porosity and density were influenced by the CaO and MgO dopants. Open porosity of 47% for undoped materials decreased to 39% for MgO and 36% for CaO, and accordingly the density increased. The conductance change *versus* RH for thick film sensors was one order of magnitude less due to a decrease in sensitivity under 40% RH. The authors pointed out that this is attributable to the deficiency of pores with diameter under 300 Å. Later, thick film humidity sensors of nanocrystalline Ba_1−x_Sr_x_TiO_3_ (x from 0 to 1 with increments of 0.2) were synthesized by the stearic acid gel method by Li and co-workers [304]. By testing the sensors in the humidity range of 11%–98% RH, the authors reported these nanocrystalline thick films showed lower resistivity rather than those made with conventional composites, and therefore could have good humidity sensing application prospects. Moreover, simplicity and cost-effectiveness were counted as advantages of this method compared with the typical sol-gel method.

In other work, a Ba_0.6_Sr_0.4_TiO_3_-MgTiO_3_ composite ceramic was prepared from BST and magnesium chloride (MgCl_2_) precursors. The molten salt MgCl_2_ was added to BST as a doping agent of the humidity-sensing catalyst. The sintered compounds were characterized in the humidity range of 5%–92% RH, resulting in a five orders of magnitude decrease in DC resistivity, and good humidity sensitivity [305]. Humidity sensing properties and the conduction mechanism of a thick film resistive type humidity sensor based on potassium (K^+^) doped nanocrystalline lanthanum cobalt ferrite oxide (LaCo_0.3_Fe_0.7_O_3_) materials has been assessed in the range of 11%–95% RH. DC and AC electrical characterization confirmed that electrons, protons, cations (H^+^ and K^+^ ions) and also molecular polarization are responsible for the charge transport in different humidity ranges [306]. In a newer work, the influence of substitution of different ions at the A site has been assessed. The electrical humidity sensitivity properties of nickel ions substituting for barium ions in porous barium tin oxide (Ba_1−x_Ni_x_SnO_3_, x = 0, 0.1, 0.2 and 0.5) have been studied by Doroftei *et al*. The single phase pellet specimens (pure BS and nickel substitution of x = 0.1) were sensitive to humidity within 22% and 75% RH, while secondary phase containing samples (x > 0.1) were sensitive to higher humidity ranges (22%–98% RH;. For the Ba_0.5_Ni_0.5_SnO_3_ sample (x = 0.5), the resistance changed by more than four orders of magnitude, and showed the fastest response among the others. The specific characteristics of this sample could be due to a fine nanostructure (∼250 nm) and the presence of a higher volume of open pores (47%) [307].

### 5.3. Humidity Sensors of Organic/Inorganic Hybrid Composites (Polymer/Ceramic)

In recent years there has been a growing contribution by the usage and evolution of composites prepared from organic/inorganic substances for humidity sensing applications. The preparation methods offer the possibility of preparing numerous potential compounds, which enable the synthesis and foundation for nanostructure sensitive films/condensed pellets [308–310]. Nanoparticles (NPs) have attracted much interest owing to their notable chemical, physical, magnetic, electronic and mechanical attributes, in addition of their bulk/surface catalytic activities and feasible utilization in electrochemical sensing applications, e.g., humidity and gas sensors. The advantages and disadvantages of organic polymers and ceramics for humidity sensing applications were described in the last sections.

Since, one of the unique properties of ceramic systems is the strong association of chemical and physical adsorption of water vapour molecules with the surface properties, thus in ceramic sensors the sensitivity and response time are principally governed by the surface morphology, pore volume, shape and size distributions. Moreover, good mechanical properties count as specific properties of ceramics. The reported problem of ceramic humidity sensors is the need for periodic heating to remove the contaminants such as dust and oils [46]. However most of the ionic type ceramic sensors have been developed without any heater and work at room temperature. The other matter which should be taken into account in the preparation of humidity-sensitive ceramics is the wise choose of the firing temperature to get the optimum interaction of water vapour and grain necks [311].

Polymeric sensitive materials have been adequately studied as bulk/film humidity sensors with respect to changes in their specific electrical conductance or dielectric permittivity upon interaction with H_2_O at room temperature as resistive or capacitive devices. Organic polymer-based sensors generally provide cost-effectiveness, simplicity of preparation and processing, choice diversity and acceptable sensitivity, although they suffer from long term drift/stability, low water-durability and accuracy, especially at high humidity, limitation of operation in harsh and chemical environments, and weak adhesion to polymeric substrates, and therefore show slow response times and short shelf lives. Furthermore, these sensors are unable to operate under high temperature conditions.

Bearing in mind the abovementioned issues, one that could be universally appropriate is the hybrid primary element. Lately, organic/inorganic compounds have gained wide attention and they are classified as a new group of sensing elements. This popularity is a result of their improved properties, as they possess the superior features of both groups. The organic/inorganic hybrids can be classified into five groups: metal/polymer, inorganic salt or acid/polymer, salt or acid/metal oxide, metal/metal oxide/polymer, and metal oxide/polymer [312–316]. The first four groups have been previously discussed in Section 5.1. In this Section metal oxide/polymer hybrid composites will be exclusively described, with some examples.

To examine the humidity sensing influence of tungsten oxide (10, 20, 30, 40 and 50 wt%) on polyaniline conducting polymers, such materials have been synthesized via a vigorous stirring method by Parvatikar *et al.* [317]. From the XRD pattern it has seen that the formed composite indicate a crystalline nature due to the observed triclinic peak of WO_3_. The pellet samples of PANI/WO_3_ (50 wt% WO_3_ in PANI) were tested under 10% to 95% relative humidity and showed a linear response, even at low humidity. Tandan *et al.* synthesized iron oxide-polypyrrole (Fe_3_O_4_-PPY) nanocomposites to form a polymerized solution using an emulsion polymerization method by means of vigorous stirring and investigated them as humidity sensitive materials [318]. The compound structure, sensitivity behaviour and conductivity value were greatly influenced by the amounts of PPY, and the resulting polymer composites showed lower resistivity compared to pure PPY, therefore meeting the criteria for being humidity sensing elements.

Novel ceramic/polymer (TiO_2_ nanoparticles/polypyrrole) and ceramic/polymer/inorganic crystalline salt (TiO_2_ nanoparticles/polypyrrole/poly-[3-(methacrylamino)propyl] trimethyl ammonium chloride) resistive humidity sensors were fabricated by Su *et al.* from composite thin films on a flexible polyester substrate through *in situ* photo-polymerization of precursors [309]. To enhance the composite sensitivity and film flexibility on the substrate, PMAPTAC was added to the TiO_2_ NPs/PPy composite thin films. By characterization of the sensors at 30% to 90% RH, it was found that the sensors made of the TiO_2_ NPs/PPy/PMAPTAC composite thin films showed the highest sensitivity, least hysteresis and greatest linearity. In a newer work by Sun *et al.* a hybrid composition inorganic/polymer humidity sensitive porous thin film of titanate and polystyrene sulfonic acid sodium salt (TiO_2_/NaPSS) has been fabricated by the dip-coating method onto an alumina substrate [319]. By testing the sensors over different relative humidity ranges (11%–95% RH), they have found several advantages as follows: the TiO_2_/NaPSS hybrid films have shown higher sensitivity, better linearity and quicker response/recovery times in all the RH ranges, and further the inorganic component (TiO_2_) could be easily distributed in the composite film with narrow hysteresis characteristic. Most of these composites exhibit improved humidity sensing behaviour rather than unalloyed polymers. High mechanical strength, water resistivity, quicker response time factors, smaller particle size and reduced hysteresis are among the key improvements.

As a brief summary it can be concluded that the majority of the sensors mentioned throughout this section, including ceramics and hybrid-based sensors, simultaneously exhibit both ionic and electronic conduction mechanisms. So the conduction by both ions and electrons (with ions being the dominant carriers) is responsible under high moisture atmospheric conditions, whilst in a low moisture environment the dominant charge carriers are electrons [320]. Therefore, at present, a commonly acknowledged sensing mechanism at room temperature is the electronic-ionic type. Humidity behaviours can be sequential in different regions:

- At low RH levels, electronic conduction based on the electron donation from water molecules is the dominant responsible mechanism.
- At medium RH levels, based on the number of physisorbed layers in different intervals, therefore electrostatic fields are due to first chemisorbed layer, both electronic and ionic conduction are responsible with the dominant transition being from electronic to ionic conduction mechanism.
- At high RH levels, ionic conduction based on proton hopping between water molecules is the dominant responsible mechanism.

## 6. Capacitive Type Humidity Sensors

The typical configuration of capacitive humidity sensors can be either a sandwiched structure with two electrode surfaces on each side, or an interdigitated structure with comb electrodes, like resistive RH sensors, such that the dielectric polymer film is deposited in between [47,105]. Several capacitive RH sensors have been designed and produced on interdigitated gold, platinum or silver electrodes with this platform incorporating organic polymer thin films or porous ceramics such as alumina, perovskites, and porous silicon based on printing deposition or coating techniques onto a ceramic substrate [321,322].

**Figure 10.**
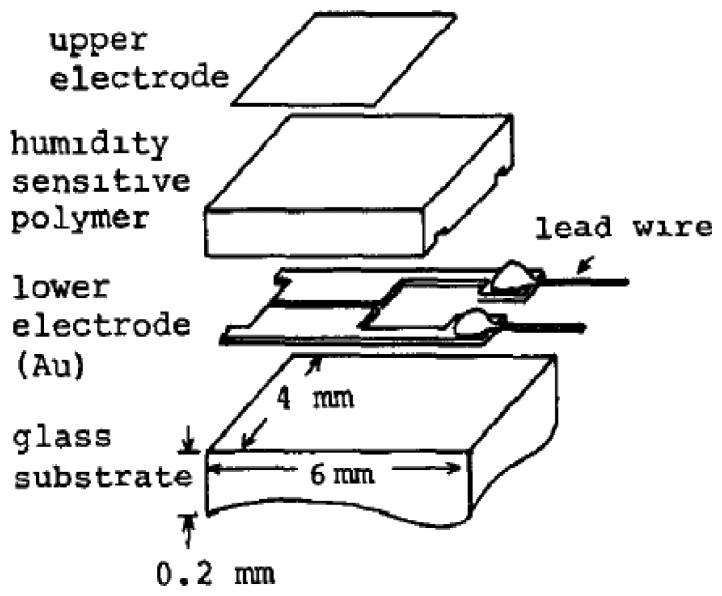
Configuration of ‘Humicape’ humidity sensor.

The parallel plate structure typically comprises two metal electrodes which are deposited on the substrate and coated with a thin film layer of a dielectric polymer or a porous ceramic metal oxide. As an upper electrode, the top of the sensing surface is coated with a thin layer of evaporated gold to protect it from ambient contamination or dust and help ensure better condensation. In the sandwich configuration, the upper porous electrode is always a water vapour permeable film [323]. A capacitive-type thin film humidity sensor called ‘Humicape’, was developed by Vaisala in Finland, and widely used in radiosonde applications and other humidity measurement instruments [47]. The sensor configuration is shown in Figure 10.

The structure was comprised of twin bottom electrodes which were attached to a glass substrate by indium evaporation. The thin film sensing layer of cellulose acetate was applied on top of that. Finally gold was evaporated as the upper electrode on top of the polymer layer. The thickness of the gold layer was around 10–20 nm and it was porous enough to allow water vapour transport through it. The sensor capacitance was changed from 45 to about 70 pF in various frequency ranges for RH range of 0% to 100% and it showed a rapid response of approximately 1 s to reach 90% of output value.

Both the coplanar types of the capacitive principle can be implemented on typical substrates of ceramic, glass or silicon. Capacitive humidity sensors respond to humidity variations by varying their dielectric permittivity, and this variation is directly proportional to the ambient vapour changes. The typical capacitance variability of the films is 0.2–0.5 pF for a 1% RH change, while this value is between 100 and 500 pF at 50% RH for the bulk capacitance at room temperature [324]. These sensors can function at high temperature ambient up to 200 °C and are fully recoverable from condensation [325]. Today, capacitive RH sensors represent more than 75% of the available humidity sensors on the market [326] and are widely applicable in the commercial, industrial and weather telemetry fields.

Currently several manufacturers offer new parallel plate configuration designs for capacitive relative humidity sensors. Their general construction contains a parallel plate capacitor sandwiched with two polymer layers. The upper layer is made from a porous polymer film and acts as a mechanical filter to prevent contamination by dust, impurities and oils. The top electrode of thin film is porous platinum or gold sputtered or evaporated on the top surface of the polymer sensing membrane. The lowest layer is the bottom electrode thick-film, which is posited between the silicon substrate and sensing film. This design utilizes a single bottom metal electrode, while ordinary ones utilise two electrodes in the bottom conductor layer. Variations of this design exist without employing the porous polymer filter layer, and various electrode geometry designs are available [327].

### 6.1. Polymer-Based Capacitive Humidity Sensors

Polymeric capacitive humidity transducers have been widely utilized in the industrial and automation field due to their ease of coating, mass production, long time stability and wide range of possible sensing polymers such as polyimides [328,329]. Many reports have been published as to capacitive sensors which some are the parallel plate PI acid capacitor on wafer silicon substrate [330], optimized RH capacitive sensors based on interdigitated electrodes and heater elements [331], high-sensitivity MEMS-based sensors [332], capacitive sensors utilising doped ion conducting polymers [333] and thin film-based cross-linked polyimide capacitive-type humidity sensors [334,335].

The design of the capacitive RH sensors pertains strongly to variations of the sensing film dielectric constant with water uptake, therefore changing the total capacitance of the configuration. Polymer dielectrics are other category of polymers, whose physical properties such as permittivity change proportionally with the high dipole moments of water molecules, therefore this humidity change is directly detected by measuring the changes of the capacitance [336,337]. In case of the capacitive polymer sensors, the room temperature relative dielectric permittivity of the polymers is approximately 5, while that for pure water is a far larger value of around 80 (78.54), and as a result the adsorption of water vapour by polymers would cause an increase of the dielectric permittivity and, thereupon, a sensitive linear change of the capacitance [46,47]. Polyimides (PI) and cellulose acetates are examples of such polymers with relative dielectric permitivitty values ranging from 3 to 6.

It is revealed that the porosity of the humidity sensitive polymer film can be manipulated to improve the sensor responses. The tensile-stressed fracture technique on thin-film proposed by Delapierre *et al.* in 1983 generates a large number of cracks in the film and at the time appeared to be a useful method without impairing conductivity or having any destructive influences [338]. A porous chromium electrode was evaporated under conditions such that the sensitive film was tensile stressed, and these stresses caused the creation of a high volume of cracks in the film, resulting in higher water vapour permeability rates by several orders of magnitude compared to nonporous polymers. Recently in other work, the results of Yeow *et al.* [339] revealed that a miniature capacitive sensor fabricated from multi-wall carbon nanotubes (MWCNTs), can naturally form porous nano-structures with a higher sensing resolution. They found that the gain in performance can be attributed to the capillary condensation effect [339].

Organic polymer-based capacitive humidity sensors are basically made from hydrophobic materials which are partly hygroscopic [105,111]. As a principle for polymer dielectrics, the absorbed water vapour occupies the free space available between the polymer molecules, thus the dielectric permittivity of the hygroscopic polymer is linearly changed proportionally to the amount of absorbed water [49]. Capacitive RH sensors typically show several key advantages in comparison with resistive-type humidity sensors [340]. One feature can be their fast and linear response to humidity along with low hysteresis [341–343]. Cost effectiveness and operation over a wide relative humidity range are among the other features of this type [344,345]. Polyimides [346], and esters have been known to be suitable candidate polymers for capacitive humidity sensors. Polyimides are the most commonly used ones and esters such as cellulose acetate butyrate (CAB) [347–349], polymethyl methacrylate (PMMA) and polyethylene terephthalate (PETT) [350,351].

Kang and Wise have introduced a high speed capacitive humidity sensor with cylindrical structure instead of the conventional structure integrated on a polysilicon heater based on multiple polyimide columns with diameters of a few microns [352]. In this configuration the moisture can penetrate into columnar-shape polyimides circumferentially, due to the higher surface area of the film, therefore high speed adsorption occurs. In this design the recovery time was reduced due to presence of the heater.

Hysteresis is a serious drawback in capacitive humidity sensors [353] and may be caused to deformation of the polymers by water clusters and consequently influence the transducer performance. Like polymer electrolytes [354], for polymer dielectrics in capacitive sensors the cross-linking method can be a solution to reduce or eliminate the hysteresis [335]. Roman *et al.* proposed that the copolymerization of PMMA with cross-linking agents will enhance the sensitivity and response time of the films and on the other hand, higher durability and lower hysteresis were achieved against acetone vapour [350].

In 1998 a capacitive humidity sensor based on a newly developed crosslinked polyimide has been fabricated by Matsuguch *et al*. The sensor contained a platinum bottom electrode which was deposited on a glass substrate by vacuum evaporation and polyimide spin-coated on top of that. After heat treatment of the film, the upper electrode was formed by evaporation of a gold layer on the film surface. The sensor was tested under different conditions and the electrical capacitance changed linearly over 10%–90% RH with a hysteresis of less than 1% RH, and in addition the sensor proved to be robust to chemicals [355].

To reduce the hysteresis characteristics of polyimide-based capacitive humidity sensors, a micro capacitive humidity sensor with a modified nano-grass PI structure as a dielectric sensitive layer was fabricated by Lee *et al.* by means of oxygen plasma treatment on a polyimide surface. By examination of the new developed structures, the nano-grass sensor showed response and sensitivity of 11 s and 0.08 pF/%RH, respectively. In comparison with the simple flat type film, the response and sensitivity were improved by about 2.5 times and 8 times, respectively. Furthermore, integration of a micro heater in the effective area has led to a reduction of the dielectric membrane hysteresis [356]. Some other polymers applied for thin film capacitive humidity sensors can also be mentioned such as polyphenylacetylene (PPA) [357] and polydimethylphosphazene (PDMP) [358].

A capacitive thin film humidity sensor based on the thin layer of [bis(benzo cyclobutene)] (BCB) dielectric material (0.8 μm thick) was recently designed and configured on a flexible polyimide substrate (8 μm thick). To optimize the sensing performance, the electrode structure has been sketched based on numerical simulations. In testing at 10% to 90% RH, the sensor showed an excellent linear response (0.996) to capacitance variations [359].

### 6.2. Ceramic-Type Capacitive Humidity Sensors

Aluminium oxide (Al_2_O_3_) with a porous structure is the most applicable material for these type of sensors [360,361], according to the electron tunnelling mechanism effect inside the condensed immobile layers of water, the Al_2_O_3_ with small pore radius is very sensitive to very low humidity levels [237,362]. Among the various phases of Al_2_O_3_ only the two phases γ-Al_2_O_3_ (amorphous) and α-Al_2_O_3_ (corundum) are commonly used in humidity sensing applications; further the prior is more sensitive than the latter one owing to its higher porosity [49]. Anodization is a basic step in porous Al_2_O_3_ humidity sensor fabrication, due to its low cost and process simplicity.

Other groups of ceramic-type capacitive moisture sensors are based on perovskite metal oxides [211,323] such as strontium lanthanum titanate (Sr_1-x_,La_x_)TiO_3_ on silicon substrate [363], SrNb_x_ Ti_1−x_O_3_ [364] and BaTiO_3_ [365]. The common structure is metal-insulator-semiconductor (MIS) in which the insulator layer is SiO_2_. The (Sr_1−x_ La_x_)TiO_3_ humidity sensor reported by Li *et al.* with MIS structure consisted of a silicon substrate, aluminium evaporated interdigitated top electrodes, thick film of (1 μm) Sr_1−x_La_x_TiO_3_ and SiO_2_ as an insulator layer. The n-type semiconducting Sr_1−x_La_x_TiO_3_ ceramic film was deposited on the SiO_2_ oxide layer via argon ion-beam sputtering and annealed at 400 °C in nitrogen [363]. To further improve of humidity sensitivity characteristics of the mentioned four-layer MIS capacitors and thin film resistor performance, the B site of the composition has been substituted by niobate (Nb) to form a strontium titanate niobate film (SrNb_x_Ti_1−x_O_3_). The presented film has shown a higher grain boundary barrier which could be explained because of substitution of Ti^4+^ by Nb^5+^ in the B sites instead of Sr^2+^ by La^3+^ in the A sites, and consequently a higher activation energy [364].

Based on the parallel plate [366] or interdigitated structure [367], both resistance and capacitance variations could be measured in these materials, however in this type the measure of capacitance variations is preferable. These ceramics are mainly deposited through thick [368] or thin films [369] and followed by post-annealing to grow grains.

Highly sensitive ZnO/TiO_2_ core/shell nanorod capacitive thin film humidity sensors were fabricated on glass substrates by Gu *et al*. Shells of sol gel processed anatase titanium oxide (TiO_2_) were deposited on hydrothermally grown zinc oxide (ZnO) nanorod cores. Morphological characterization showed that the primarily formed zinc oxide nanorods were coated with anatase titanium oxide shells as a second layer. Compared with those sensors based on individual ZnO and TiO_2_, the ZnO/TiO_2_ nanocomposite (ZTNA) sensors exhibited considerably enhanced sensitivity at 95% RH (31 and 1,380 times greater than the ZnO nanorod arrays and TiO_2_ thin films, respectively). Further, the core/shell arrays' capacitance varied from 10^1^ to 10^6^ pF over the whole humidity range of 11% to 95% RH at room temperature [370].

Recently, the humidity sensing properties of two types of bismuth phosphates *i.e.*, cubic sillenite and monoclinic types synthesized through a hydrothermal method were investigated by Sheng and co-workers with respect to their capacitive characteristics [371]. Based on the humidity sensing characterization, a sensor with the cubic bismuth phosphate structure revealed linear capacitance variations of four orders, namely from 1.1 pF to 12,908 pF over the entire RH range, while the monoclinic bismuth phosphate sample showed only three orders of magnitude change from 1.2 pF to 1,097 pF in a nonlinear fashion. The conduction mechanism of the composites was reported to be based on the presence of polarizable Bi^3+^ cations incorporated with moderate PO dopants. The sensing mechanisms have been reported to be of two main types: “non-Debye” types at low RH and “proton transport” mechanisms at high RH.

Like the sensing mechanism of previous types, the pore properties of capacitive type sensors are responsible for their humidity sensing performance and influence the physisorption and chemisorption of water vapour molecules as well as capillary condensation [372].

Proton-exchange membrane fuel cells (PEMFCs) are one of the pragmatic applications of humidity sensors [373]. The ambient conditions such as humidity, temperature and air flow are highly influential on the efficiency of PEMFCs, therefore, the optimum performance of fuel cell systems can be only achieved by the recognition and control of these conditions through the design and utilization of expert sensor systems [374]. However, shortcomings of available humidity sensors for working at high temperature ambient in the presence of invariantly high humidity levels made them unsuitable for PEMFC applications, thereby motivating the need to develop and the evolution of micro-flexible resistive [375,376] and capacitive [377,378] humidity sensing systems integrated into the fuel cell systems [379,380].

Lee *et al.* were motivated to design and fabricate a home-made MEMS-based novel flexible multi-functional micro sensor integrated on a stainless steel foil substrate which was able to sense temperature, humidity and voltage. The proposed multi-functional micro sensors could be embedded in the cathode flow channel of a proton-exchange membrane fuel cell (PEMFC) to measure the variations of internal voltage, temperature and humidity. Fabricated micro sensors have benefited from numerous desirable advantages, encompassing high flexibility due to their stainless steel foil substrate, high temperature resistance, high sensitivity, tiny size, precision of measurement, and capability to operate in high moisture (high RH) conditions along with long term stability. To reduce the temperature dependence of the humidity sensor, a capacitive principle of construction based on an interdigitated structure with a polyimide sensitive film has been adopted instead of a resistive type. Furthermore, to eliminate a long time response of the micro humidity sensor, it has been integrated with a micro heater [381].

## 7. Conclusions/Outlook

The moisture sensing properties, manufacturing technologies and operating mechanisms of various humidity sensors based on applications and different sensing elements consisting of ceramic, organic polymer and hybrid composites have been classified and comprehensively described. Synthesis and preparation methods of precursor metal oxides and polymers for hygrometric applications were also explained. To provide an overall perspective, a review of state-of-the-art humidity transducers has been rendered in table form. Regarding the mechanism of sensing, a protonic conduction type has been discussed in detailed as a generally accepted mechanism in the majority of sensors (more than 85%) at room temperature. The key factors such as design configuration, structural characteristics and applied precursor quantities that influence efficiency, stability and sensitivity have been discussed.

Among all humidity sensor design configurations, the impedance- (resistive) and capacitive-based sensors are revealed to be the best suited and most popular ones in advanced applications such as laboratory research or automated industries. This popularity could be due to their ability to fulfil the general requirements such as simpler structure, free choice of sensing elements among various available types, cost, adaptability to different types of circuits, and ease of fabrication and measurement setup.

Nowadays, film-based humidity sensors are widely applied because of their advantages such as cost-effectiveness, acceptable gasketing, design flexibility and quick deposition rate. Among the various types of the humidity sensors, semiconducting metal oxide and metal oxide/polymer based sensors, dominantly produced by thick film and thin film deposition techniques, are noteworthy due to their variety of sensitive element choices, post-processing and greater response characteristics. In comparison with polymer-based thin or thick film humidity sensors, the synthesis process of the ceramics is simpler and they frequently reveal very fast response times, however polymers are lower in cost. These sensors are operational over a wide range of humidity with instrumental response characteristics, and possess the potential of being commercially available for medium to high volume demands.

Recent developed humidity sensors in both university research and industry, especially those incorporated with nano-scale elements, provide promising performance with the high impact contribution to an improvement of accuracy, reliability, and economic efficiency. However, in the real conditions of practical environments, there still remain challenges to be solved for enhancing the sensor efficiency and response characteristics. In the future outlook of the humidity sensors design process, the nanocrystalline composites incorporating ceramics and ceramic/polymer are among the most promising materials, which may be applied for humidity sensing applications due to the better performance prospects they exhibit.

## Figures and Tables

**Table 1.** The state-of-the-art of humidity sensors based on fabrication technologies and sensing materials.

**Type**	**Fabrication Technology**	**Sensing Material**			**Cost**	**Specs**

		**Electrolytes**	**Polymers**	**Ceramics**		
**Relative**	Conventional Ceramic/Semiconductor Processing	Available	Not Available	Available	Low	Simple, and Bulk Application
	Thick Film, LTCC	Not Available	Available	Available	Medium	Signal Drift
	Thin Film	Not Available	Available	Available	Medium	Hysteresis
	p-n Heterojunction	Not Available	Not Available	Available	Medium- High	In Progress

**Absolute**	Solid Moisture (Al_2_O_3_)	Not Available	Not Available	Available	Medium- Low	Versatile
	Chilled Mirror Dew/Frost Point	Not Available	Not Available	Not Available	High	Complex

**Table 2.** Examples of the different relative humidity sensors based upon the technologies and specifications.

**Specification**		**Sensing Material**				**Transduction Type**		**Sensing Mechanism**		**Reference (Resistive)**	**Reference (Capacitive)**
	
**Technology**		**Electrolyte**	**Polymer**	**Ceramic**	**Organic/Inorganic (Hybrid)**	**Resistive**	**Capacitive**	Electronic *	**Protonic**		

								**Electrolytic**			
**Conventional**	**Ceramic/Semiconductor Processing**	Not Available	Not Available	Available	Available	Available	Available	Ceramic	Ceramic	29,31,33*,34,40*,44,51,58,59, 60*,61*,62,64,69*,86,182–192, 203,206,208-211,214,215,218, 221,224,225,259–262,267–278, 297,298,301,305,307.	44,69*, 211, 224,344.

									Hybrid	308,317,318.	-

	**Lithium Chloride Electrolyte (LiCl)**	Available	Not Available	Not Available	Not Available	Available	Not Available	-	Electrolyte	47,52.	-

	**Thick Film**	Not Available	Available	Available	Available	Available	Available	Ceramic	Polymer	49,54,98,166.	321,330,340.

									Ceramic	30,32,36,48,49,95,178,207,216, 220,222,226,228,237–258, 263–266,279,280,299,300, 302–304, 306.	368.
									Hybrid		
										-	-

	**Thin Film**	Not Available	Available	Available	Available	Available	Available	Ceramic	Polymer	28,46,47,49,53,99, 105–108,113–137, 141–146,147–165,175.	46,47,49,55, 105,111,323, 325–329, 331–359.

									Ceramic	26,34,42,47,48,49,56,57, 69,91,96,100–104,179, 180,193,194,196,204,205, 212,219,227–231,245,281–296.	47,49,57,212,323,325–327, 360–367, 369–372.
									Hybrid		
										27,37,41,138–140,167–174, 309,310,312–316,319.	27,37,322.
